# Noncovalent interaction guided selectivity of haloaromatic isomers in a flexible porous coordination polymer†

**DOI:** 10.1039/d3sc03079b

**Published:** 2023-10-07

**Authors:** Rohan Jena, Subhajit Laha, Nimish Dwarkanath, Arpan Hazra, Ritesh Haldar, Sundaram Balasubramanian, Tapas Kumar Maji

**Affiliations:** a Chemistry and Physics of Materials Unit (CPMU), School of Advanced Materials (SAMat) Jawaharlal Nehru Centre for Advanced Scientific Research (JNCASR) Jakkur Bangalore-560064 India tmaji@jncasr.ac.in; b Tata Institute of Fundamental Research Hyderabad Gopanpally Hyderabad 500046 Telangana India

## Abstract

Porous, supramolecular structures exhibit preferential encapsulation of guest molecules, primarily by means of differences in the order of (noncovalent) interactions. The encapsulation preferences can be for geometry (dimension and shape) and the chemical nature of the guest. While geometry-based sorting is relatively straightforward using advanced porous materials, designing a “chemical nature” specific host is not. To introduce “chemical specificity”, the host must retain an accessible and complementary recognition site. In the case of a supramolecular, porous coordination polymer (PCP) [Zn(*o*-phen)(ndc)] (*o*-phen: 1,10-phenanthroline, ndc: 2,6-naphthalenedicarboxylate) host, equipped with an adaptable recognition pocket, we have discovered that the preferential encapsulation of a haloaromatic isomer is not only for dimension and shape, but also for the “chemical nature” of the guest. This selectivity, *i.e.*, preference for the dimension, shape and chemical nature, is not guided by any complementary recognition site, which is commonly required for “chemical specificity”. Insights from crystal structures and computational studies unveil that the differences in the different types of noncovalent host–guest interaction strengths, acting in a concerted fashion, yield the unique selectivity.

## Abstract 2

Porous, supramolecular structures exhibit preferential encapsulation of haloaromatic isomers, primarily by means of differences in the order of (noncovalent) interactions.

## Graphical abstract

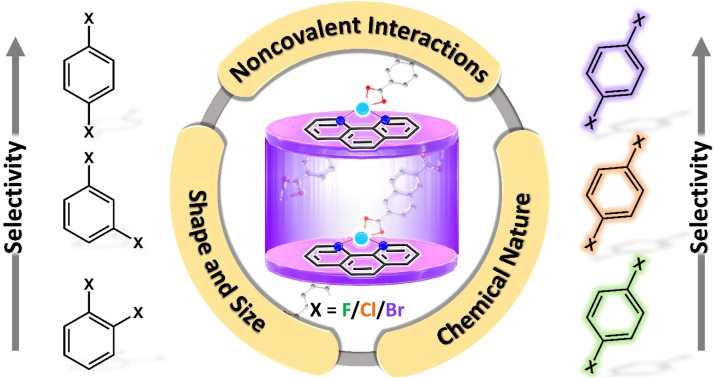

## Introduction

Dimension, shape, and chemical nature – based on any of these parameters, a mixture of chemicals can be separated using porous materials.^1–4^ To perform selective separations, new porous materials have been developed over the last two decades.^5–8^ These are made by connecting specific geometry nodes *via* dynamic crosslinking bonds.^9^ As a result, empty spaces or voids can be designed with desirable dimensions and chemical functionalities. By judiciously combining metal ions and complementary organic linkers, porous coordination polymers (PCPs)^10^ and metal–organic cages^11^ can be obtained; using organic linkers alone, covalent organic frameworks (COFs),^12^ conjugated microporous polymers (CMPs),^13^ and organic cages^14^ can also be designed. With the advantages of easy structural tailorability, it is evident that pores of specific shapes and sizes can be made for desired chemical separations.^15,16^ This scheme works in a straightforward manner, but in the case of a mixture of molecules with very similar physical and chemical properties, selectivity decreases drastically. This is because of the fact that molecules are discriminated on the basis of dimension, shape, or chemical nature, not all three at once. Molecular recognition by a combination of all three is non-trivial to design and realize.

**Scheme 1:**
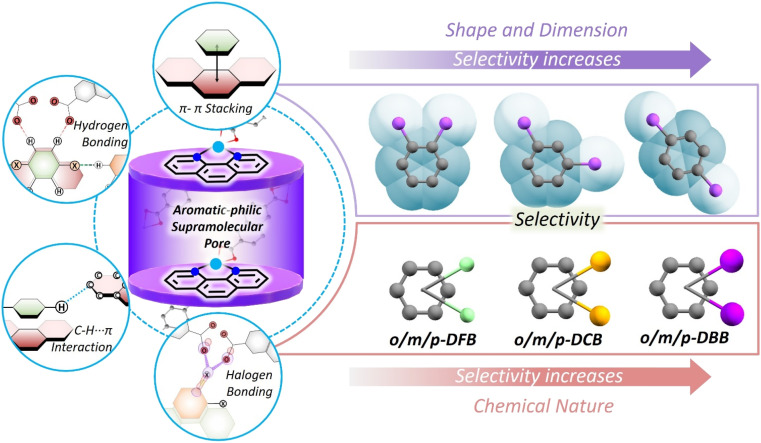
A schematic representation of selective encapsulation of haloaromatic isomers in a flexible porous coordination polymer; left panels show the supramolecular aromatic-philic pores created between the *o*-phen units of two adjacent 1D-chains of the PCP and the supramolecular interactions affecting the packing of various guest molecules in the nanopore. Right panels show the geometrical and chemical nature driven selectivity for the guest molecules.

One way to introduce a more advanced recognition system in these porous materials is by allowing the pores to adjust to the guest molecules. The adjustment will depend on the dimension, shape as well as chemical nature of the guests. Among PCPs, a sub-category, *i.e.*, flexible or soft PCPs exists.^17^ This class of PCPs is known to adapt its pore structure to the environment.^18,19^ These structures have flexible metal-nodes or geometrically flexible organic linkers. These features allow the structure to undergo contraction, expansion, or some specific distortion^20–29^ in response to changes in temperature and pressure, or upon filling of guest molecules in the voids.^30,31^ These structural responses are neither easy to predict nor to tune. Many previous reports on flexible PCPs illustrate structural changes, such as breathing, net movement in the entangled PCPs and hinge-like motion of 3D nets.^19,32–34^ In this work, we have realized selectivity by exploiting noncovalent interactions, acting in a concerted way, in a flexible PCP (Scheme 1). Noncovalent interactions, such as hydrogen bonding, aromatic π–π, and C–H–π interactions are much weaker compared to coordination or covalent bonds, and hence allow geometric flexibility, and can also be chemical-nature specific. We explore this possibility in a flexible porous coordination polymer containing a supramolecular pocket with tunable void space.^35,36^

To test our hypothesis, we chose a library of positional haloaromatic isomers containing planar benzene rings. Among these selected isomers, dihalobenzenes are widely used as organic solvents, raw materials and intermediates for pharmaceuticals, pesticides and dye industries.^37–43^ The recognition and separation of these are of high value and remain a grand challenge.^44,45^ The dihalobenzene isomers have (i) different geometries due to the difference in positions of halogen substitution and (ii) different halogens (fluorine: F, chlorine: Cl, and bromine: Br) having different polarizabilities and sizes.^46,47^ Considering these differences, we have tested the separation capabilities in a Zn-based PCP {[Zn(*o*-phen)(ndc)]·(DMF)}, having aromatic-philic supramolecular pockets (*o*-phen: 1,10-phenanthroline, ndc: 2,6-naphthalenedicarboxylate, DMF: dimethylformamide).^48^ The preference for aromatic planar molecules is due to the unique pore-environment created by two sandwiching *o*-phen. Such an arrangement offers optimum space to host planar guest molecules like benzene, toluene, xylene, and substituted anilines.^48,49^ The combination of π⋯π, C–H⋯π, and hydrogen bonding interactions in this PCP yields unique molecular preferences. By performing *in situ* crystallization experiments on isomer-mixtures and single crystal structure determination, it was revealed that the host PCP prefers *para*-isomers (1,4-substituted) by geometric consideration and Br-substituted guests by chemical preference. As a result, ∼100% selective encapsulation was observed for the *para*-dibromo isomer from the mixture of *para*/*ortho* dibromo isomers, while for the *para*-difluoro- and dichloro-isomers, the host showed lower selectivities. The cooperativity between geometric and chemical preference improves the selectivity factor, and in the following section, we unveil the key parameters responsible, through structural insights of guest-encapsulated crystal structures and first-principles computational studies.

## Results and discussion

**Fig. 1:**
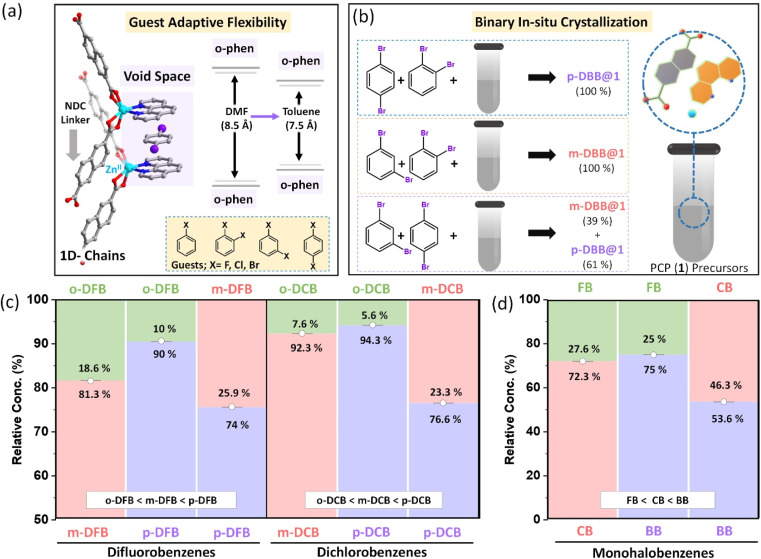
(a) Illustration of a nanospace created between the *o*-phen of adjacent 1D chains of PCPs, (b) experimental procedure for *in situ* crystallization of binary aliquot mixtures of dibromobenzenes (DBB), and the right panel shows reaction vessel containing PCP precursors *i.e.*, 2,6-NDC, *o*-phen, and z Zn(NO_3_)_2_⋅6H_2_O in DMF,bar diagram showing relative amounts of isomers encapsulated after binary *in situ* crystallization of positional isomers of (c) dihalobenzene and (d) monohalobenzenes. [note: the components of each binary mixture for difluorobenzenes (DFB), dichlorobenzenes (DCB) and monohalobenzenes are mentioned at the top and bottom of the respective plots along with their relative concentrations].

A large number of flexible PCPs are available;^50^ we have selected one that can specifically host substituted benzene molecules. The selected PCP is constructed by linking Zn(ii)-*o*-phen node with an NDC linker. This 1D structure, as illustrated in Fig. S1,† self-assembles into a 3D porous structure held together by noncovalent interactions (reported elsewhere,^48^ see Fig. S1†). This creates void spaces between a pair of *o*-phen of neighboring chains, and hosts solvent molecules (DMF or aromatic molecules) as shown in Fig. 1a. In earlier work, it has been observed that the PCP is porous in nature with intrinsic guest induced flexibility. The removal of the guest leads to the shrinkage of the pores, which reverts back to it's original porous structure on adsorption of the guest, confirmed by *in situ* adsorption-PXRD measurements.^48^ In the presence of DMF, the *o*-phen⋯*o*-phen distance is ∼8.5 Å, while for toluene, it reduces to about 7.5 Å.^48^ Hence the pore size is adjusted in accordance with the chemical nature of the guests. Similar changes were observed earlier, in the case of xylene isomers, *i.e.*, structural adjustments induced by the geometry of the guest.^49^ The above features of the PCP prompted us to study its selectivities for haloaromatic isomers.

Two classes of halo-substituted benzene isomers are considered for separation using the selected PCP: ternary or binary mixtures of (i) monohalobenzenes, namely, fluorobenzene (FB), chlorobenzene (CB), and bromobenzene (BB) and (ii) dihalobenzenes with halogen substitutions in *ortho*, *meta* and *para* configurations. For the latter class, only mixtures containing positional isomers of a given dihalobenzene, namely, difluorobenzene (DFB), dichlorobenzene (DCB), and dibromobenzene (DBB) are used. The PCP is assessed for a more challenging task of separating isomers of a given dihalobenzene having similar physicochemical properties rather than those with different halogen substitutions whose boiling points differ substantially. To probe the selectivities we have devised an *in situ* multicomponent crystallization-based experiment, as illustrated in Fig. 1b. In this method, the PCP precursors and the mono-/di-halobenzene isomer mixtures are mixed together to crystallize in the first set of experiments, and for each of the three dihalobenzenes, we tested *ortho*/*meta*, *ortho*/*para*, and *meta*/*para* mixtures (see ESI section S3 and Table S1, S2† with nomenclature guest@1). After the *in situ* crystallization experiments, the crystals were washed thoroughly and digested for characterization using ^1^H-NMR experiments (see ESI section S4 and Fig. S3–S7†). The relative amounts of the isomers estimated by the ^1^H-NMR experiments are shown in Fig. 1b–d (see Fig. S3–S7†). For all three dihalobenzenes, a common preference of *ortho* < *meta* < *para* is observed. However, there are two noticeable differences in the case of DBB compared to DFB or DCB: (i) the *ortho*/*meta* and *ortho*/*para* selectivity factors are nearly ∼99 (see ESI Table S3†) for DBB and (ii) the *meta*/*para* selectivity factor is lower for DBB (Fig. 1b). Similar experiments were carried out using the ternary mixtures, *i.e.*, with mixtures of *ortho*, *meta*, and *para* isomers of each dihalobenzene (See Fig. S6†). The selectivity trend is *ortho* < *meta* < *para*, consistent with the binary mixture experiments (Fig. S3–S6†). It is worth noting that *ortho* isomers of DCB and DBB are exclusively rejected in their respective series. These experiments demonstrate that the PCP shows (i) a geometric preference for the *para* isomers and (ii) relative concentrations as well as selectivity factors, in general, improve with larger halogen size. To confirm this chemical preference, we have carried out *in situ* multi-component crystallization experiments for the mixture of fluorobenzene (FB), chlorobenzene (CB), and bromobenzene (BB) (see Fig. S7(a–c)†). The preference order is FB < CB < BB (Fig. 1d). This observation is in accordance with the results observed for binary and ternary dihalobenzene isomer mixtures. Such a concurrent geometry and chemical preference is unique. Additionally, the PCP is capable of selectively adsorbing halobenzene isomers, which has been demonstrated from the liquid-phase batch reactions using activated PCPs (see section S5, Fig. S14–S18 and Table S4†). The relative concentrations and selectivity values precisely match with the binary/ternary *in situ* crystallization results. Furthermore, at a relatively lower concentration of *p*-DBB in the mixtures of *p*-DBB and *o*-DBB, *p*-DBB is preferentially adsorbed by the PCP, as can be seen from Fig. S18.†

To understand the unique selectivity properties of the PCP, it is important to gain insights into the host–guest noncovalent interactions. To this end, we have carried out (i) crystal structure determination of the individual isomers encapsulated in the supramolecular pores using SCXRD experiments, (ii) periodic-density functional theory (DFT) calculations of the determined crystal structures to quantify the binding energies and study the extent of noncovalent interactions, and (iii) estimation of the enthalpy of guest binding (Δ*H*) using differential scanning calorimetry (DSC) experiments.

**Fig. 2:**
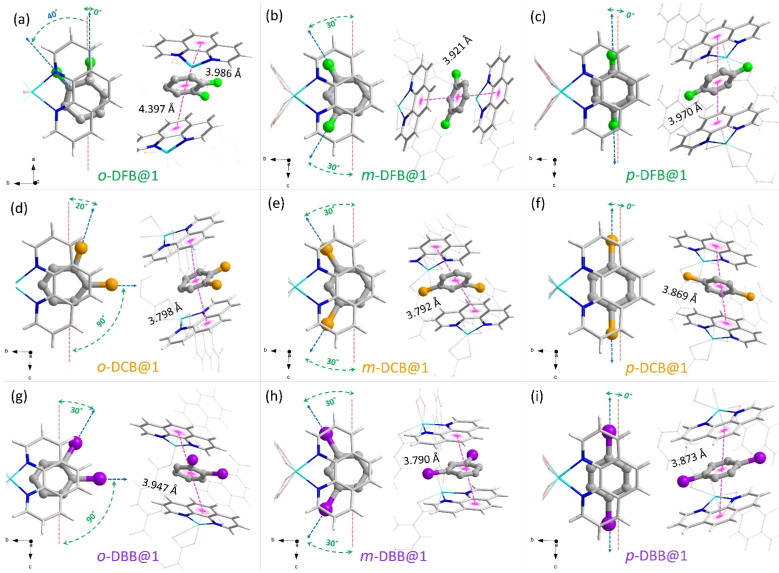
Guest molecule encapsulation in a “dynamic clip” in their respective guest@1 crystal structures determined using SCXRD experiments. Top and side views of the guest encapsulated cavities are shown on the left and on the right sides of each panel, along with their respective centroid-to-centroid distances from the sandwiching *o*-phen rings. (a–c) Difluorobenzenes, *i.e.*, *o*-DFB@1, *m*-DFB@1 and *p*-DFB@1, (d–f) dichlorobenzenes, *i.e.*, *o*-DCB@1, *m*-DCB@1 and *p*-DCB@1, (g–i) dibromobenzenes, *i.e.*, *o*-DBB@1, *m*-DBB@1 and *p*-DBB@1. Color scheme—framework: hydrogen (white), carbon (silver), nitrogen (blue), oxygen (red), and zinc (cyan). Guests: carbon (gray), fluorine (green), chlorine (orange), and bromine (purple). Hydrogen atoms of the guest molecules are not shown for clarity. The guest molecules are represented by a thicker “ball-and-stick” representation.

We could synthesize and characterize the individual monohalobenzene and dihalobenzene isomer-encapsulated PCP single crystals (guest@1) (CCDC no. 2266788–2266798). In all cases, the guest molecules are sandwiched between the *o*-phen rings, as illustrated in Fig. 2 (see ESI section S6†). This spatial geometry allows for facile π–π stacking to stabilize the guest@host crystal structures. In Fig. 2a–c, the three isomers of DFB encapsulated in the supramolecular pocket are shown with details of the orientation and π–π stacking. For *o*-DFB@1, the C–F bond is oriented inwards with a twist angle of 40° (angle between the *a*-axis and the C–F bond) and the guest is not sandwiched symmetrically by the two *o*-phen rings with centroid-to-centroid distances of 3.986 and 4.397 Å. This is because the aromatic planes of the guest and *o*-phen are not parallel (Fig. 2a). However, for both *m*-DFB@1 and *p*-DFB@1, the two centroid-to-centroid distances are identical and are shorter than that for *o*-DFB@1, yielding distances of 3.921 and 3.970 Å, respectively. This clearly indicates that the π–π stacking interactions are weaker for the *ortho* isomer and hence it is least preferred (in accordance with the observed selectivity). The spatial geometries of the *ortho* isomers of DCB and DBB are slightly different compared to the case of DFB, as shown in Fig. 2d and g. The C–Cl/C–Br bonds are projected outward with twist angles of 20° (Cl) and 30° (Br) and the aromatic planes of the guest and *o*-phen are nearly parallel. However, for the *meta* and *para* isomers, spatial positions are very similar to those of any of the halogen substitutions, except for small differences in the centroid-to-centroid distance (see Fig. 2). For the monohalobenzenes, no distinct difference in the guest spatial geometry was observed (Fig. S19†). These insights provide a qualitative explanation of the guest encapsulation and spatial geometry. The origin of preferential encapsulation of guests however, remains unclear.

**Fig. 3:**
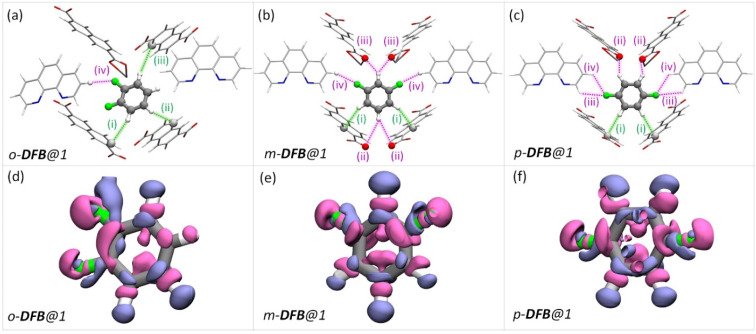
(a–c) The guest encapsulated cavities of (a) *o*-DFB@1, (b) *m*-DFB@1, and (c) *p*-DFB@1, respectively. *o*-phen rings sandwiching the guest molecules are omitted for clarity. The colored dashed-lines represent the type of supramolecular interactions (green and magenta lines represent C–H⋯π and hydrogen bonding interactions, respectively). Details of atom names, bond lengths, and angles associated with each interaction are shown in Table S18.† (d–f) Electron density differences in the vicinity of the guest in a cavity of (a) *o*-DFB@1, (b) *m*-DFB@1, and (c) *p*-DFB@1, respectively. Violet and magenta surfaces represent electron density gain and loss of magnitude 5.0 × 10^−4^ a.u., respectively. The guest encapsulated cavities shown here were obtained from the guest@1 super cells optimized using periodic-DFT. The color scheme for the atoms is identical to that in Fig. 2.

**Fig. 4:**
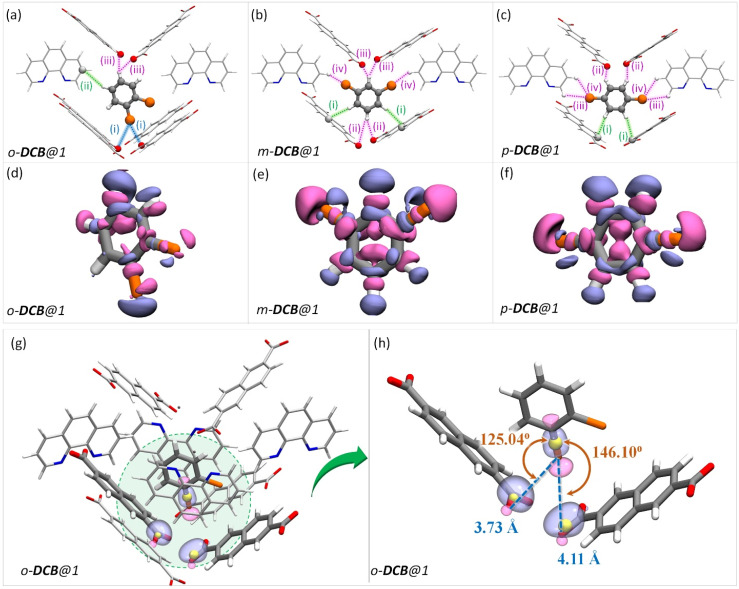
The guest encapsulated cavities of (a) *o*-DCB@1, (b) *m*-DCB@1, and (c) *p*-DCB@1, respectively. *o*-phen rings sandwiching the guest molecules are omitted for clarity. Details of the atom names, bond lengths and angles associated with each interaction are shown in Table S19.† (d–f) Electron density differences in the vicinity of the guest in a cavity of (a) *o*-DCB@1, (b) *m*-DCB@1, and (c) *p*-DCB@1, respectively. The coloring scheme used in this figure is identical to that in Fig. 2. (g and h) Reproduction of Fig. 4(a) with maximally localised Wannier functions (MLWFs; violet and magenta surfaces) and maximally localised Wannier function centres (MLWFCs; yellow spheres) for the C–Cl σ-bond (of *o*-DCB) and electron-pair in π-bonds (PB) of the framework C=O1 bonds. Violet and magenta surfaces represent MLWFs of opposite phases with an isovalue of 5.0 × 10^−2^ a.u., with an exception of the σ-bond of C–Cl which is shown for an isovalue of 1.5 × 10^−1^ a.u. ∠C–Cl⋯PB are 125.04° and 146.10°, confirming that the π-bond electrons indeed interact with the σ-hole of the chlorine atom. All the guest encapsulated cavities shown in this figure were extracted from the respective guest@1 supercells of periodic DFT calculations, post cell optimization.

**Fig. 5:**
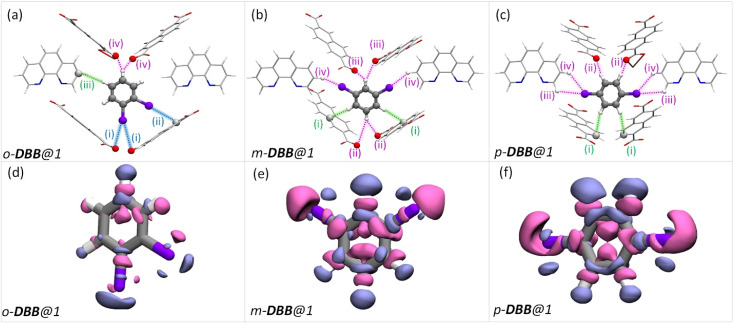
Dibromobenzene encapsulated cavities of 1. Each panel is analogous to the corresponding panels in Fig. 4(a–f), but bromine is in the place of chlorine atoms. Color schemes, the protocol for obtaining the structures, *etc.* are again identical to that in Fig. 4(a–f).

To rationalize the observed order of guest preference, a quantification of the host–guest interaction strengths and specific host-guest interactions are required. To this end, we have employed periodic-density functional theory (DFT) calculations using the Gaussian plane wave^51^ method implemented in Quickstep^52,53^ of the CP2K-7.1 package^53,54^ (see the ESI for details). First, periodic-DFT-based cell optimizations (CO) of supercells of all the experimentally determined crystal structures of guest@1 were performed. Later, the optimized structures were used to generate electron density difference maps from single point energy calculations (see section S8†) as a visual aid to identify the regions of PCP which interact with the guest molecules (Fig. 3–5 and S26†). Among DFB isomers, *ortho*-DFB exhibits four possible interactions – one hydrogen (HB) bonding and three C–H⋯π interactions (see Fig. 3a). For each of the *meta* and *para* isomers, eight interactions are seen, as illustrated in Fig. 3b and c. Although the types and number of interactions are the same for the *meta* and *para* isomers, *p*-DFB and *m*-DFB show prominent gain in electron density around hydrogen atoms in their respective crystal structures (Fig. 3e and f). The calculated binding energies (ESI section S8, Table S17†) are −124.90, −124.70 and −108.60 kJ mol^−1^ for *para*, *meta*, and *ortho* DFB, respectively, and this order is in accordance with the experimental observation (see ^1^H NMR results and the selectivity order, Fig. 1c).

In the case of DCB, the *ortho* isomer exhibits two types of interactions: two of its hydrogen atoms have close contact with the framework, one forming a C–H⋯π and the other forming a HB with NDC and carboxylate oxygen of the framework, respectively. In the case of *meta* and *para* isomers, the interactions are more in number, as shown in Fig. 4. However, a noticeable difference in the case of *o*-DCB is the presence of a halogen bonding interaction with the PCP. When a halogen atom, ‘X’, is bonded to another group ‘R’, X suffers a depletion of electron density near its polar region with respect to the R–X bond, called a “σ-hole”. A halogen bond emerges when an electron-rich group ‘A’ (A = lone pair, π-electrons, *etc.*) interacts with the electron-depleted region of X. Such a setting demands ∠R−X⋯A = 180°.^55^ This geometry-based criterion for halogen bonding identification is a necessary condition but not a sufficient one, especially for the case of guests confined within MOF/PCP pores.^56^ The electronic structure, however, definitively reveals the presence of such interactions. Hence, to elucidate this specific interaction (blue-dashed line in Fig. 4a), we have calculated maximally localized Wannier functions (MLWFs), and their corresponding centers – maximally localised Wannier function centers (MLWFCs),^56–58^ MLWFs (violet surfaces) and MLWFCs (yellow spheres) of C=O1 (Fig. 4g–h) show that the π-electrons of the double bond face the C–Cl axis.

Such an orientation indicates the interactions between the C–Cl σ-hole and the electron-rich π-clouds of the C=O1 bond. This observation explains the difference in the spatial orientation of *o*-DCB and *o*-DFB. However, this additional halogen bonding is not substantial enough to affect the selectivity trend noted earlier. Furthermore, among the stacked-benzene dimers, the “slip” geometry is more favored than the “face-on” geometry, and the energy difference is about 4 kJ mol^−1^.^59,60^ Hence, *p*-DCB, the only DCB isomer having a slip w. r. t. the *o*-phen rings is more stabilized in the cavity than the other two isomers. Furthermore, binding energy calculations corroborate the selectivity preference of *ortho* < *meta* < *para*, with binding energies of −134.40, −142.70, and −144.00 kJ mol^−1^, in the same order (see Table S17 and Fig. S27†). For the DBB isomers, the nature of interactions is very similar to that of DCB. The *o*-DBB isomer, again, shows halogen bonding interactions; however the total possible interactions are still fewer compared to those of *meta* or *para* isomers. This is illustrated in Fig. 5. A noticeable difference is the change in the halogen bonding angle; for Br the halogen bond angle C–O–Br/Cl is larger, close to the ideal 180° (see Fig. S25†). This indicates stronger halogen bonding for Br, and this is in accordance with the polarizability trend of the halogens.^55^*o*-DBB also has a small region of electron density in the vicinity of the other halogen atom (Fig. 5d) alluding to another halogen bonding interaction not present for *o*-DCB in its crystal structure. Irrespective of this, the selectivity trend remains identical to that of DFB or DCB with binding energies of −138.50, −150.70, and −151.90 kJ mol^−1^, for *o*-, *m*-, and *p*-DBB, respectively (ESI, Table S17, Fig. S27†).

We noted that due to the nonpolarizable nature of fluorine,^55^*o*-DFB does not participate in halogen bonding interactions with the PCP. Consequently, the position of *o*-DFB within the cavity is distinctively different from those of either *o*-DCB or *o*-DBB. On the other hand, the corresponding orientations of *meta* and *para* isomers within the PCP cavity are similar for all three dihalobenzenes, and so are the guest–framework interactions. However, differences in the binding energies among *meta* (or *para*) isomers of dihalobenzenes are significant for dihalobenzenes with different halogen substitutions (Table S17†). Halogen atoms of *meta* or *para* dihalobenzenes interact *via* hydrogen bonding with the framework. Due to the increase in polarizability with halogen size, the strength of hydrogen bonding interactions is in the order F < Cl < Br;^55^ thus the binding energies are in the order *p*-DFB < *p*-DCB < *p*-DBB (similarly for *meta* isomers) (ESI, Table S17†). In addition to differences in the hydrogen bonding strengths, other factors can also bring about differences in the binding energies. Thus, the PCP not only discriminates positional isomers of dihalobenzenes, but also on the basis of the halogen or, simply, based on the chemical nature.

Additional confirmation for the selectivity was obtained through differential scanning calorimetry by experimental quantification of the guest binding enthalpy (Δ*H*). We observed that the order of binding enthalpy is identical to the selectivity trend obtained in the *in situ* crystallization experiments. For the dihalobenzenes, from *ortho* to *para*, the binding enthalpy increased linearly. However, the energy difference between the different halogen isomers is negligible, *e.g.*, the enthalpy values for the *ortho* DFB, DCB, and DBB are 69.53 ± 4.39, 76.86 ± 2.28 and 71.92 ± 1.14 J g^−1^ (see Fig. S20†). The difference due to the presence of different halogen atoms becomes clear by comparing the binding enthalpy values of the monohalobenzenes. These are 39.55 ± 1.81, 103.65 ± 2.35, and 116.90 ± 4.50 J g^−1^ for FB, CB, and BB, respectively (Table S16†). The order of binding enthalpies measured by DSC experiments and the binding energies obtained by periodic-DFT calculations are identical for various guest molecule encapsulations in the PCP.

## Conclusion

In conclusion, we have demonstrated selective encapsulation of halobenzene isomers using a flexible porous coordination polymer. The current work showcases the prominence of concerted supramolecular interactions for the optimal and selective placement of guest molecules in a geometrically ordered position. This resulted from the directionally specific, short-range noncovalent interactions inside the nanospace. The adaptive nature of the PCP preferentially sorts *para*- > *meta*- > *ortho* dihalobenzene geometrical isomers and *bromo-> chloro-> fluoro*- in the case of monohalobenzenes. This preference is experimentally demonstrated by crystallization and separation experiments. All the experimental results were further corroborated by extensive computational studies such as *ab initio* cell optimizations, electron density difference mappings and periodic density functional theory (DFT) analyses. Additionally, theoretical binding energies and experimentally determined enthalpies unequivocally supported the order of selectivity obtained by the crystallization experiments. This unique preference of the supramolecular framework is attributed to the strength of the noncovalent interactions inside the pocket, but not to the type or number of interactions. The findings presented here can be a tool guide to design more advanced supramolecular systems, which can perform easy recognition and separation of challenging isomers and isotopes.

## Data availability

All associated data are in the ESI† or deposited with CCDC (2266788–2266798).

## Author contributions

R. J. and T. K. M. designed the concept of this work. R. J. performed major experiments and data analysis. S. L. assisted in the data analysis. N. D. performed all the theoretical calculations. A. H. assisted in the crystal structure refinements. R. H., S. B., and T. K. M. assisted in the writing and editing of the manuscript. All authors contributed to the preparation of the manuscript.

## Conflicts of interest

There are no conflicts to declare.

## Supplementary Material

SC-014-D3SC03079B-s001

SC-014-D3SC03079B-s002
