# Supplementary material for: Noncovalent interaction guided selectivity of haloaromatic isomers in a flexible porous coordination polymer
Source: Chem Sci. 2023 Oct 7;14(43):12321–30. doi: 10.1039/d3sc03079b (PMC10631220; doi:10.1039/d3sc03079b)
Supplement: SC-014-D3SC03079B-s001 [file SC-014-D3SC03079B-s001.pdf]

Electronic Supplementary Material (ESI) for Chemical Science.
This journal is © The Royal Society of Chemistry 2023

Supporting Information

Noncovalent Interaction Guided Selectivity of Haloaromatic Isomers in a Flexible Porous Coordination Polymer

Rohan Jena[a], Subhajit Laha[a], Nimish Dwarkanath[a], Arpan Hazra[a], Ritesh Haldar[b*], S. Balasubramanian[a*], Tapas Kumar Maji[a*]

[a] Chemistry and Physics of Materials Unit, School of Advanced Materials, Jawaharlal Nehru Centre for Advanced Scientific Research, Jakkur, Bangalore-560064, India.

[b] Tata Institute of Fundamental Research Hyderabad, Gopanpally, Hyderabad 500046, Telangana, India

E-mail: tmaji@jncasr.ac.in

Contents

S1. Materials

S2. Synthetic procedure

S3. Binary/ternary component in-situ crystallization

S4. NMR Analyses (Relative concentrations and selectivity factors)

S5. Batch Reactions using activated PCP

S6. SCXRD Analyses of individual guest solvate crystals

S7. DSC Analyses

S8. Ab-initio cell optimization and periodic density functional theory calculations (DFT)

S1. Materials: All the reagents employed were commercially available and used as provided. Zinc nitrate hexahydrate, 2,6-naphthalenedicarboxylicacid, 1,10-phenanthroline, N,N-dimethylformamide (anhydrous), fluorobenzene, chlorobenzene, bromobenzene, ortho/meta/para-dichlorobenzenes, ortho/meta/para-dibromobenzenes and ortho/meta/para-difluorobenzenes were purchased from Sigma Aldrich chemicals.

S2: (a) Synthesis of PCP (1) [Zn(ndc)(o-phen)]·(DMF): Synthesis of **1** was carried out according to the previously reported methodology[1]. 2,6-naphthalenedicarboxylic acid (0.022 g, 0.1 mmol) and 1,10-phenanthroline (0.020 g, 0.1 mmol) were dissolved in 5 mL of N,N-dimethyl formamide (DMF) solvent and mixed well. 0.030 g (0.1 mmol) of $Zn(NO_3)_2·6H_2O$ was added to the ligand solution and sonicated and transferred to a 15 mL screw capped glass vial, which was then sealed properly using Teflon tape and kept in an hot air oven at 120 ^{0}C for 36 h. Crystals of **1** were isolated and washed with fresh DMF before the single crystal X-ray diffraction measurement to check structural details.

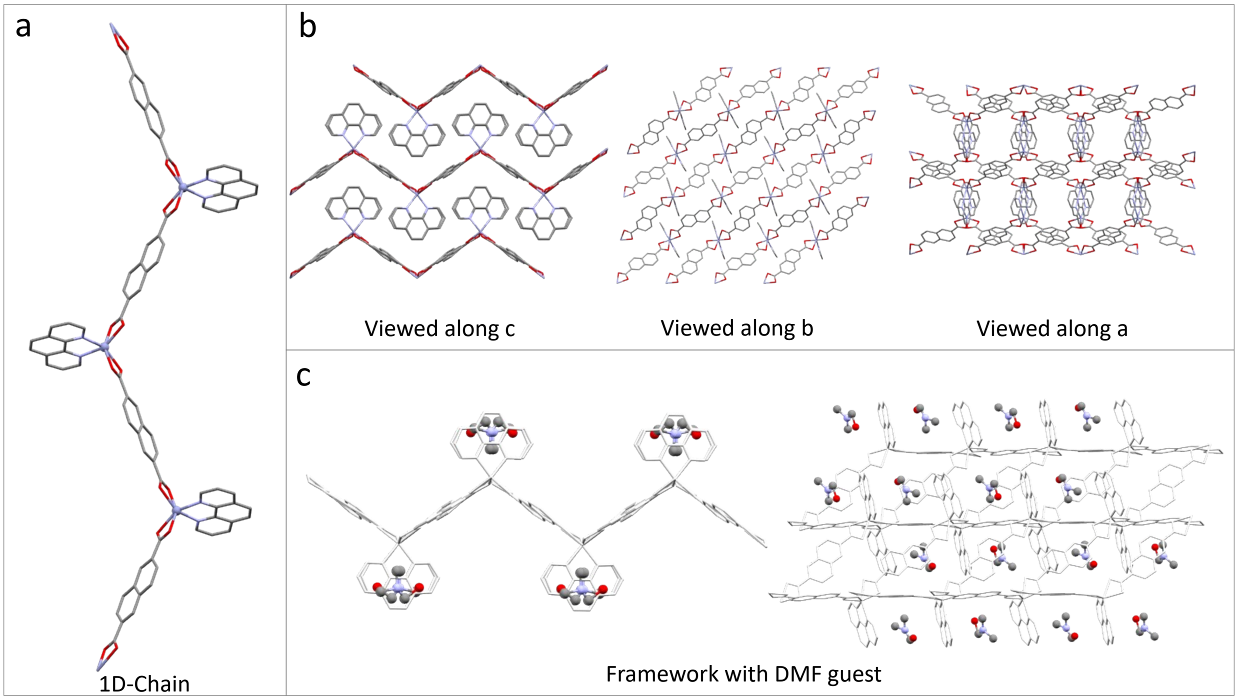

Fig. S1: (a) 1D-Chain of [Zn(o-phen)ndc], (b) PCP framework viewed along a, b, c directions, (c) PCP structure in presence of guest (DMF) molecules.

S2: (b) Syntheses of [Zn(ndc)(o-phen)]·(Guest):

[Zn(ndc)(o-phen)]·(**o-DFB**)], [Zn(ndc)(o-phen)]·(**m-DFB**)], [Zn(ndc)(o-phen)]·(**p-DFB**)], [Zn(ndc)(o-phen)]·(**o-DCB**)], [Zn(ndc)(o-phen)]·(**m-DCB)**], [Zn(ndc)(o-phen)]·(**p-DCB**)], [Zn(ndc)(o-phen)]·(**o-DBB**)], [Zn(ndc)(o-phen)]·(**m-DBB**)], [Zn(ndc)(o-phen)]·(**p-DBB**)], [Zn(ndc)(o-phen)]·(**FB**)], [Zn(ndc)(o-phen)]·(**CB**)], [Zn(ndc)(o-phen)]·(**BB**)]

2,6-naphthalenedicarboxylic acid (0.022 g, 0.1 mmol) and 1,10-phenanthroline (0.02 g, 0.1 mmol) were dissolved in 5 mL of N,N-dimethylformamide (DMF) solvent and mixed well. 0.030 g (0.1 mmol) of $Zn(NO_3)_2{\cdot}6H_2O$ was added to the ligand solution and sonicated for 10 min. 1 mL of aromatic guest was added to this clear solution before the sealed glass vial was kept in an hot air oven at 120 ^{o}C for 36 h. Good quality light yellow color crystals were isolated and washed with fresh DMF before taking for single-crystal X-ray diffraction measurement.

S3. Binary/Ternary in-situ crystallization

Synthesis protocol: Binary in-situ crystallization

In-situ crystallization of o-DFB and m-DFB in PCP: Equimolar mixture of o-DFB (6.25 mmol) and m-DFB (6.25 mmol) were taken in a 20 ml screwcap vial. To this mixture, precursors of PCP (1) were added, i.e., 2,6-naphthalenedicarboxylic acid (0.1 mmol) and 1,10-phenanthroline (0.1 mmol), 5 mL of dimethyl formamide (DMF) and $Zn(NO_3)_2{\cdot}6H_2O$ (0.1 mmol). The resulting solution was sealed properly using Teflon tape, sonicated for 10 mins and kept at 120 ^{0}C for 48h.

The product crystals were kept at RT for cooling and then washed with plenty of DMF to wash away unreacted residual species from the crystal surface and dried in open air for 24h. The obtained crystals were named as *o-/m-**DFB**@1*.

Similarly**,** other combination of binary component crystals were obtained by following the similar procedure, with various halobenzene mixtures listed below.

All the obtained crystals are listed in **Table S1 and S2**.

Table S1: Nomenclature of crystal samples obtained from binary in-situ crystallization of respective isomers/compounds.

		Mixture Components		Resulting Crystal
DXB	***DFB***	*o-**DFB***	*m-**DFB***	*o-/m-**DFB**@1*
		*o-**DFB***	*p-**DFB***	*o-/p-**DFB**@1*
		*m-**DFB***	*p-**DFB***	*m-/p-**DFB**@1*
	DCB	*o-**DCB***	*m-**DCB***	*o-/m-**DCB**@1*
		*o-**DCB***	*p-**DCB***	*o-/p-**DCB**@1*
		*m-**DCB***	*p-**DCB***	*m-/p-**DCB**@1*
	DBB	*o-**DBB***	*m-**DBB***	*o-/m-**DBB**@1*
		*o-**DBB***	*p-**DBB***	*o-/p-**DBB**@1*
		*m-**DBB***	*p-**DBB***	*m-/p-**DBB**@1*
XB	***FB CB BB***	***FB***	***CB***	***FB/CB**@1*
		FB	***BB***	***FB/BB**@1*
		CB	***BB***	***CB/BB**@1*

Synthesis protocol: Ternary in-situ crystallization

Similar protocol has been followed as mentioned for binary component in-situ crystallizations, except for the number of halobenzene compounds. All three isomers (ortho-, meta- and para-) for dihalobenzenes (DFBs or DCBs or DBBs) were mixed together in equimolar concentrations before solvothermal crystallization.

For monohalobenzenes, all three compounds (i.e., fluorobenzene (FB), chlorobenzene (CB) and bromobenzene (BB)) were mixed together in equimolar ratios.

Table S2: Nomenclature of crystal samples obtained from ternary in-situ crystallization of respective isomers/compounds.

		Mixture Components			Resulting Crystal
DXB	***DFB***	*o-**DFB***	*m-**DFB***	*p-**DFB***	*o-/m-/p-**DFB**@1*
	DCB	*o-**DCB***	*m-**DCB***	*p-**DCB***	*o-/m-/p-**DCB**@1*
	DBB	*o-**DBB***	*m-**DBB***	*p-**DBB***	*o-/m-/p-**DBB**@1*
XB	***FB, CB, BB***	***FB***	***CB***	***BB***	***FB/CB/BB**@1*

S4. NMR Analysis of binary/ternary crystals

Quantification of the guest by NMR analysis: The obtained crystals were first analyzed by single crystal X-ray diffraction measurements to detect and quantify the different guest molecules trapped inside the nanospace. The crystals obtained from the binary aliquot mixtures exhibited similar unit cell parameters in comparison to individual crystal systems (**Table S5-S15**). Also, the crystal structure showed the most stabilized isomer in the nanospace, whose diffraction pattern overshadowed the other isomer present in minor quantity. To avoid the errors during the quantification of guests in the crystals enclosing more than one positional isomer, we have performed ^{1}H NMR analysis.

^{1}H NMR of digested crystals: To tackle this challenge, ^{1}H-NMR was considered to be a more convenient tool to detect all the isomers present within the encapsulated PCP crystals and further relative quantifications could be estimated made by integrating the suitable proton signals. The detailed procedure for ^{1}H-NMR analyses of binary/ternary *in-situ* crystals are mentioned as follows and the corresponding spectra are shown in next sections.

Digestion of crystal samples: 5-10 mg of each crystal samples were taken in a 2mL microcentrifuge tube, followed by the addition of 15µL of conc. HCl. Further 1000 µL of DMSO-d6 was added to the crystal-HCl mixture. The tube containing the mixture was then sonicated till the all the crystals dissolve to produce a homogenous solution. The resulting solution was then transferred into a NMR tube and further used for ^{1}H NMR analysis (Schematic is shown in **Fig. S2**).

Fig. S3-S7 shows the high-resolution NMR spectra of digested crystals obtained from the aliquots of binary mixtures. For better visualization, the small region between 6.5 to 8.0 ppm is shown and the respective isomers are represented alongside with each NMR spectrum. The relative percentage of one guest over others was calculated by normalizing the integrated peak areas and the number of protons by considering a single guest per formula unit. (Full range NMR spectra are shown in **Fig. S8-S13**)

Relative Concentrations:

$$Relative\ Concentration\ (R) = \frac{A_i}{\sum_{i=1}^{n} A_i} \times 100$$

Where, A_i: Peak area in ^{1}H NMR normalized with no. of protons in each halobenzene

Note: The peak positions and integrated peak areas were assigned to respective halobenzenes present in either binary or ternary in-situ crystals, by comparing the ^{1}H NMR spectra of individual pure halobenzene compounds. In case, the peak positions are overlapped, then peak areas were assigned by considering the non-overlapped peaks and thereafter correlating/adjusting/subtracting the remaining expected peak areas from overlapped region.

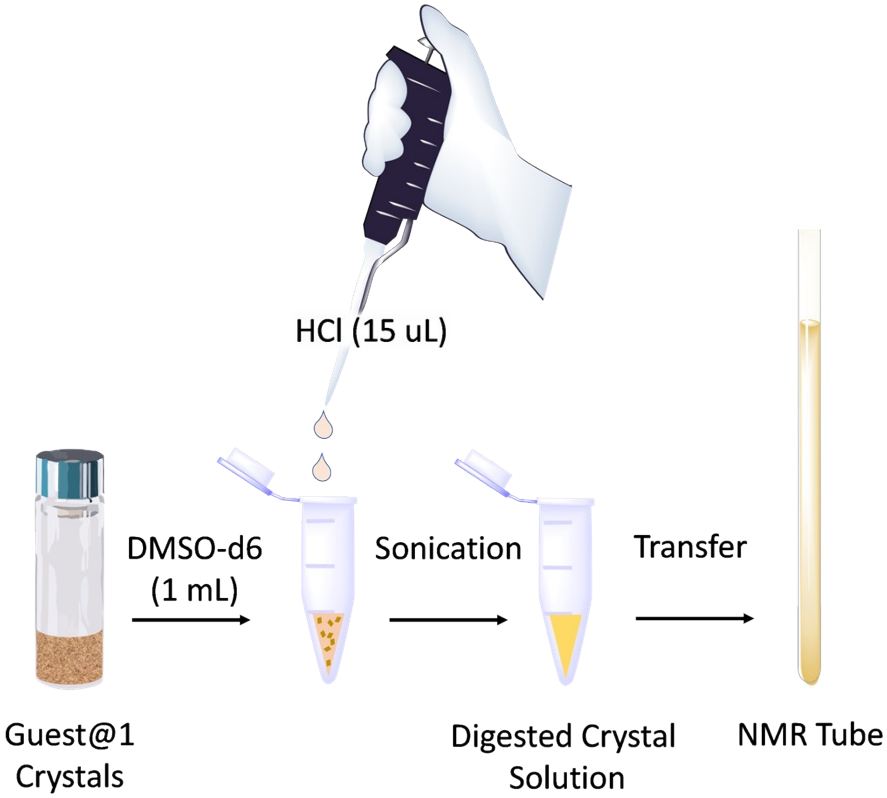

Fig. S2: Schematic of NMR analysis of binary/ternary crystals via digestion method

NMR Analysis: Relative Concentration (R) calculation

Binary in-situ crystallization: Difluorobenzene series (DFBs)

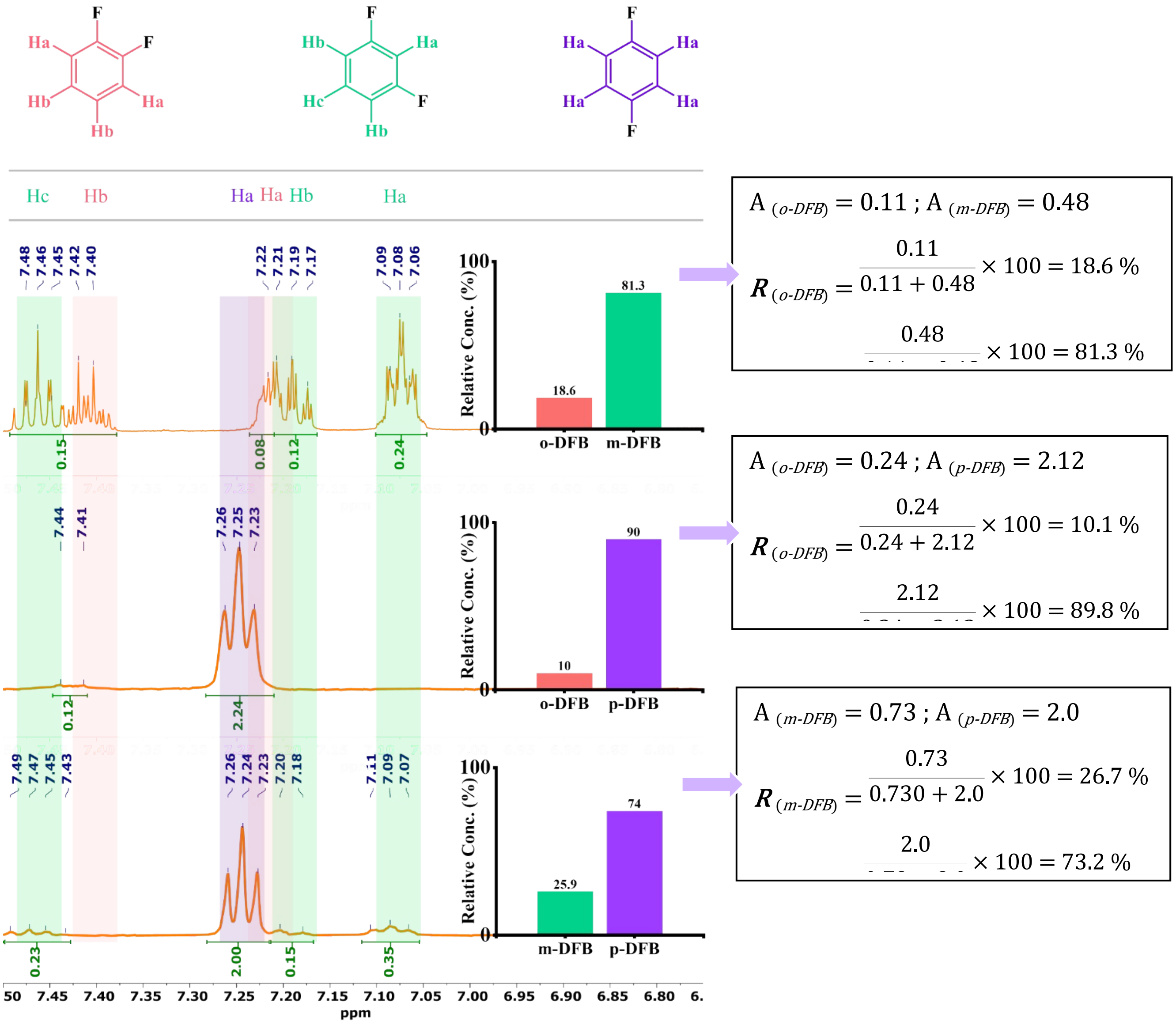

Fig. S3: ^{1}H NMR spectra of digested crystals obtained from binary in-situ crystallization of difluorobenzene isomers.

NMR Analysis: Relative Concentration (R) Calculation

Binary in-situ crystallization: Dichlorobenzene series (DCBs)

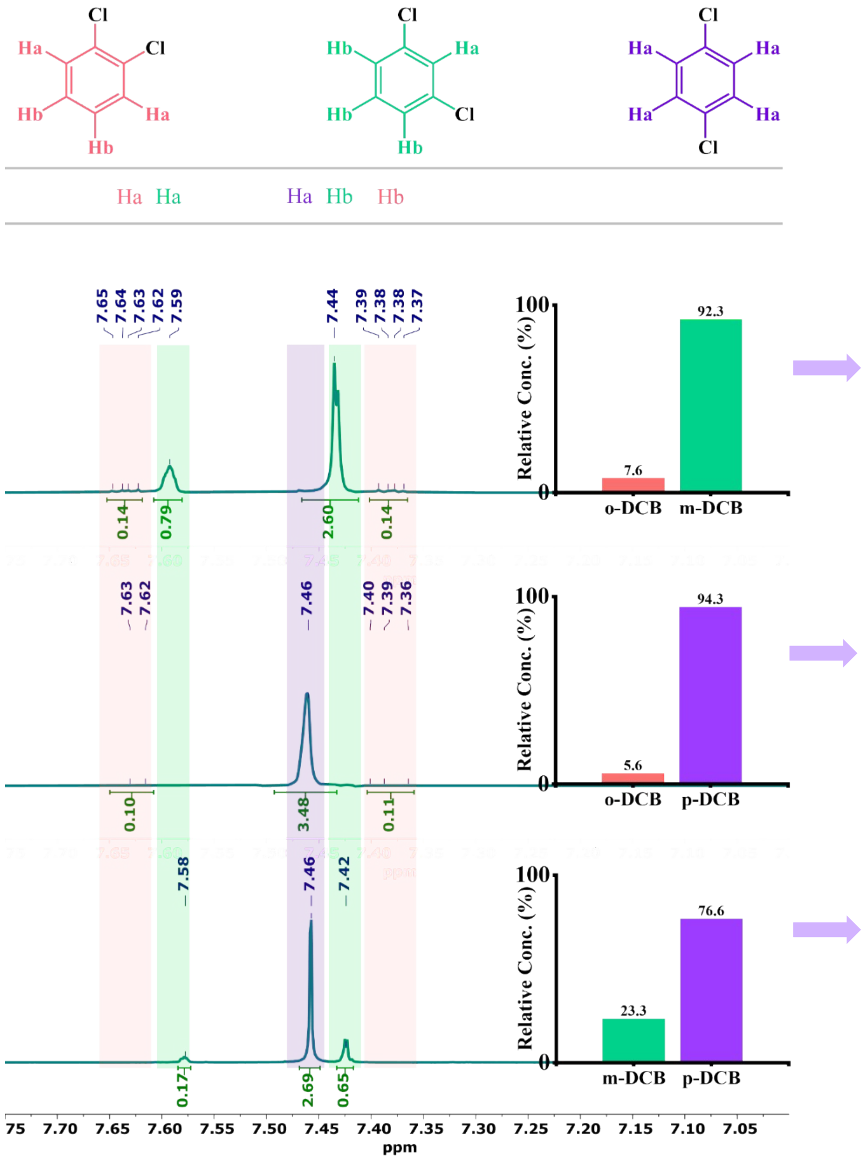

$A_{(o\text{-}DCB)} = 0.28$; $A_{(m\text{-}DCB)} = 3.39$

$$R_{(o\text{-}DCB)} = \frac{0.28}{0.28 + 3.39} \times 100 = 7.6\ \%$$

$R_{(m\text{-}DCB)} =$ [illegible]

$A_{(o\text{-}DCB)} = 0.21$; $A_{(p\text{-}DCB)} = 3.48$

$$R_{(o\text{-}DCB)} = \frac{0.21}{0.21 + 3.48} \times 100 = 5.6\ \%$$

$\frac{3.48}{[illegible]} \times 100 = 94.3\ \%$

$A_{(m\text{-}DCB)} = 0.82$; $A_{(p\text{-}DCB)} = 2.69$

$$R_{(m\text{-}DCB)} = \frac{0.82}{0.82 + 2.69} \times 100 = 23.3\ \%$$

$\frac{2.69}{[illegible]} \times 100 = 76.6\ \%$

Fig. S4: ^{1}H NMR spectra of digested crystals obtained from binary in-situ crystallization of dichlorobenzene isomers.

NMR Analysis: Relative Concentration (R) Calculation

Binary in-situ crystallization: Dibromobenzene series (DBBs)

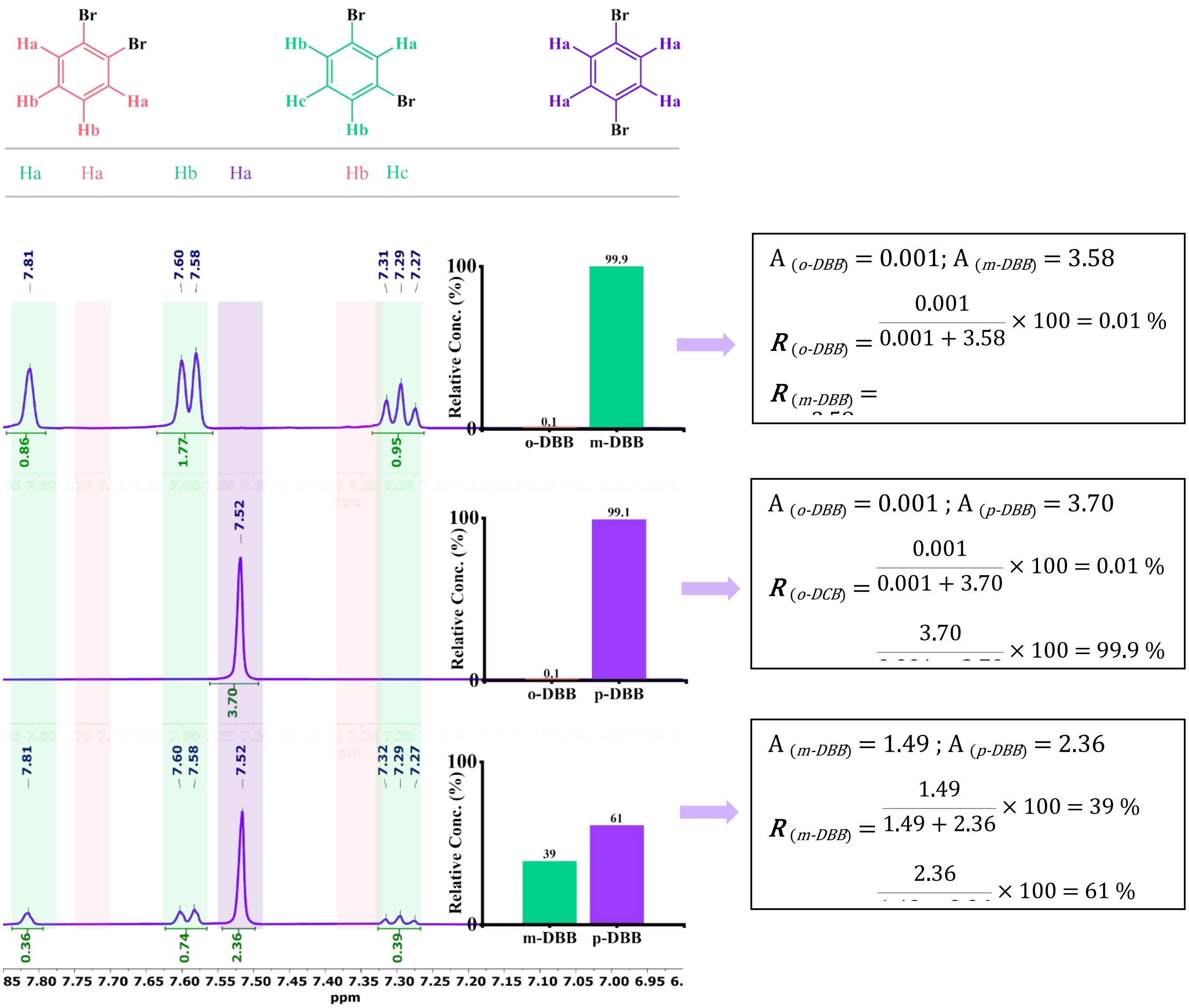

Fig. S5: ^{1}H NMR spectra of digested crystals obtained from binary in-situ crystallization of dibromobenzene isomers.

NMR Analysis: Relative Concentration (R) Calculation

Ternary in-situ crystallization: Dihalobenzene series (*DFBs, DCBs* and *DBBs*)

(a)

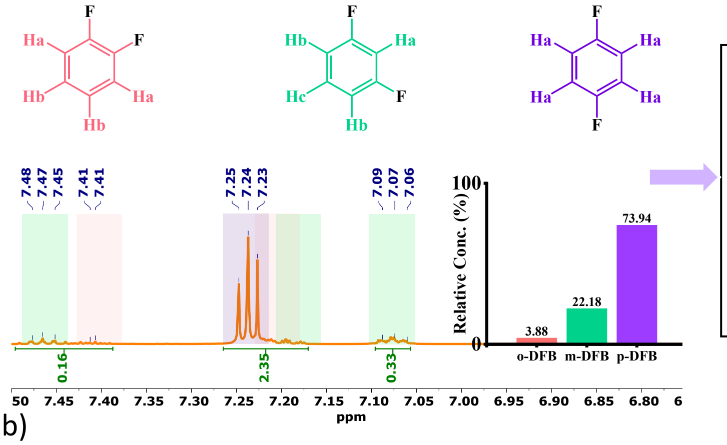

$$A_{(o\text{-}DFB)} = 0.02,\ A_{(m\text{-}DFB)} = 0.66,\ A_{(p\text{-}DFB)} = 2.16$$

$$\boldsymbol{R}_{(o\text{-}DFB)} = \frac{0.02}{0.02 + 0.66 + 2.16} \times 100 = 0.7\ \%$$

$$\boldsymbol{R}_{(m\text{-}DFB)} = \frac{0.66}{0.02 + 0.66 + 2.16} \times 100 = 23.2\ \%$$

2.16

(b)

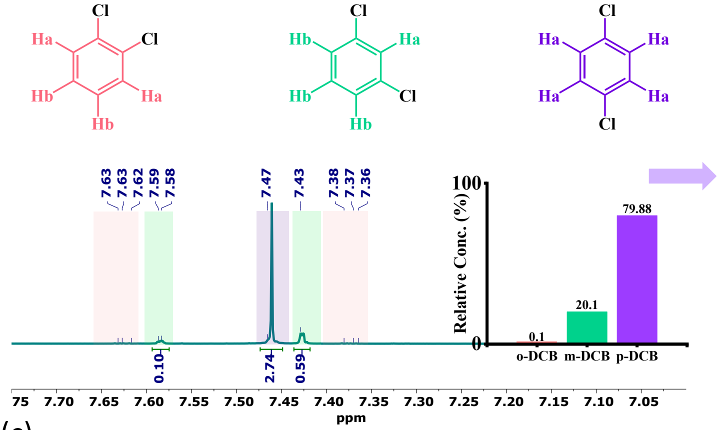

$$A_{(o\text{-}DCB)} = 0.01,\ A_{(m\text{-}DCB)} = 0.69,\ A_{(p\text{-}DCB)} = 2.74$$

$$\boldsymbol{R}_{(o\text{-}DCB)} = \frac{0.01}{0.01 + 0.69 + 2.74} \times 100 = 0.1\ \%$$

$$\boldsymbol{R}_{(m\text{-}DCB)} = \frac{0.69}{0.01 + 0.69 + 2.74} \times 100 = 20.1\ \%$$

2.74

(c)

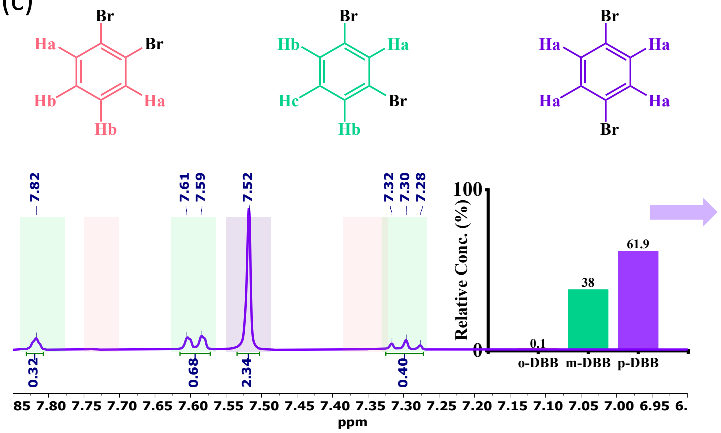

$$A_{(o\text{-}DBB)} = 0.01,\ A_{(m\text{-}DBB)} = 1.4,\ A_{(p\text{-}DBB)} = 2.34$$

$$\boldsymbol{R}_{(o\text{-}DBB)} = \frac{0.01}{0.01 + 1.4 + 2.34} \times 100 = 0.1\ \%$$

$$\boldsymbol{R}_{(m\text{-}DBB)} = \frac{1.4}{0.01 + 1.4 + 2.34} \times 100 = 38\ \%$$

2.34

Fig. S6: ^{1}H NMR spectra of digested crystals obtained from ternary in-situ crystallization of (a) difluorobenzene, (b) dichlorobenzene, (c) dibromobenzene isomers.

NMR Analysis: Relative Concentration (R) Calculation

Binary and ternary in-situ crystallization: Monohalobenzenes (*FB, CB* and *BB*)

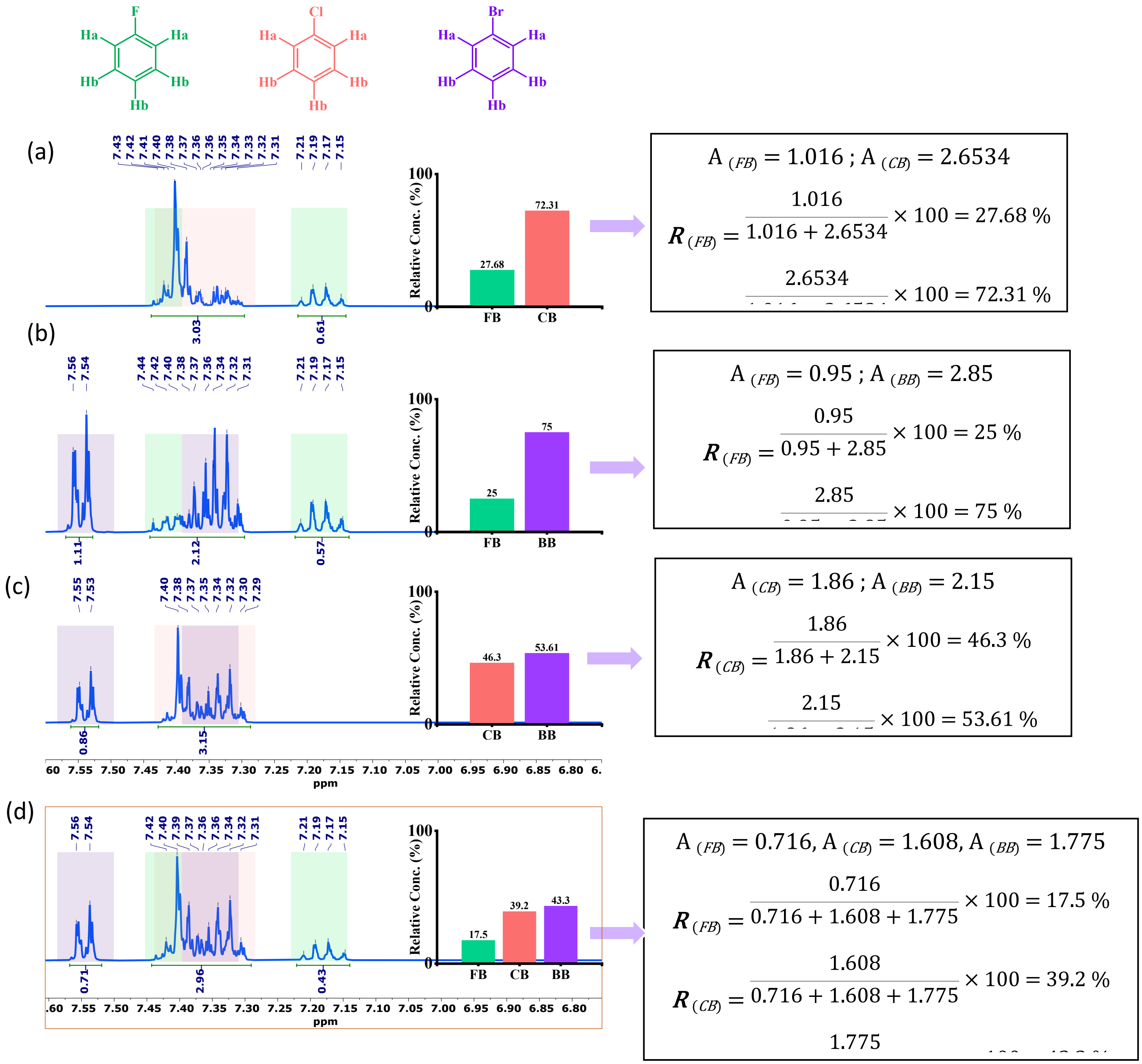

Fig. S7: ^{1}H NMR spectra of digested crystals obtained from **(a-c)** binary in-situ crystallization of monohalobenzenes, **(d)** ternary in-situ crystallization of monohalobenzenes

Calculation of Selectivity Factor

For Binary Mixtures

$$S_A = \frac{Relative\ Conc.\ of\ A}{Relative\ Conc.\ of\ B} \qquad S_B = \frac{Relative\ Conc.\ of\ B}{Relative\ Conc.\ of\ A}$$

For Ternary Mixtures

$$S_{A\ (BC)} = \frac{2 * Relative\ Conc.\ of\ A}{Relative\ Conc.\ of\ B + Relative\ Conc.\ of\ B}$$

Table S3: Selectivity factors calculated from respective relative concentrations.

**Binary Components**	**Selectivity**
o-DFB + m-DFB	$S_{m\text{-}DFB}$ = 4.36
o-DFB + p-DFB	$S_{p\text{-}DFB}$ = 9
m-DFB + p-DFB	$S_{p\text{-}DFB}$ = 2.86
o-DCB + m-DCB	$S_{m\text{-}DCB}$ = 12.14
o-DCB + p-DCB	$S_{p\text{-}DCB}$ = 16.84
m-DCB + p-DCB	$S_{p\text{-}DCB}$ = 3.29
o-DBB + m-DBB	$S_{m\text{-}DBB}$ = 99.9
o-DBB + p-DBB	$S_{p\text{-}DBB}$ = 99.9
m-DBB + p-DBB	$S_{p\text{-}DBB}$ = 1.56
**Ternary Components**	**Selectivity**
o-DFB + m-DFB + p-DFB	$S_{p\text{-}DFB}$ = 6.36
o-DCB + m-DCB + p-DCB	$S_{p\text{-}DCB}$ = 7.91
o-DBB + m-DBB + p-DBB	$S_{p\text{-}DBB}$ = 3.25
**Monohalobenzenes**	**Selectivity**
FB + CB	S_{CB} = 2.61
FB + BB	S_{BB} = 3
CB + BB	S_{BB} = 1.16

FB + CB + BB	S_{BB} = 1.53

NMR Spctra Showing PCP components along with halobenzene guests

NMR Spectra (Binary mixture of DFBs)

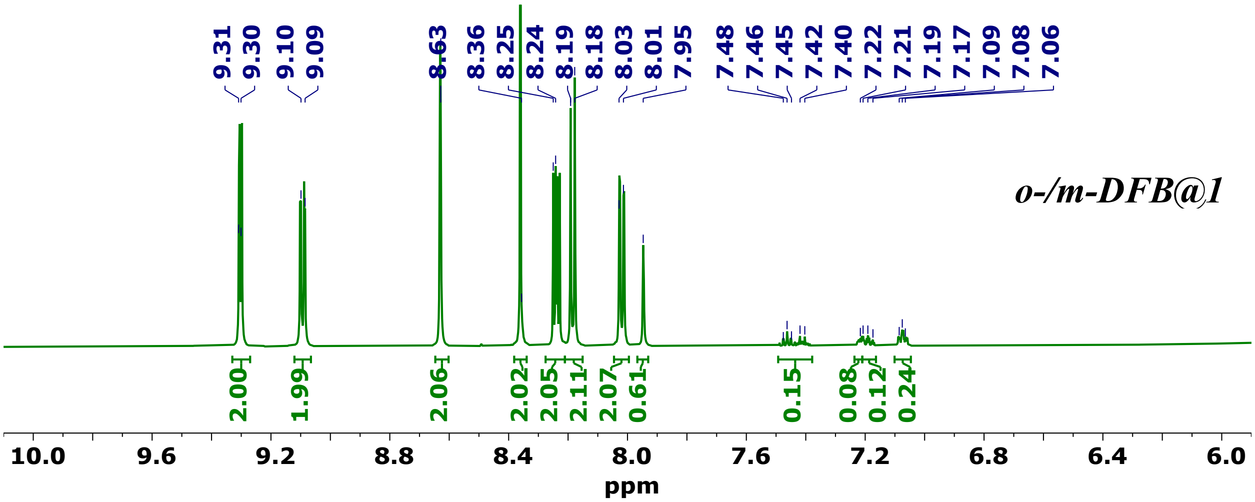

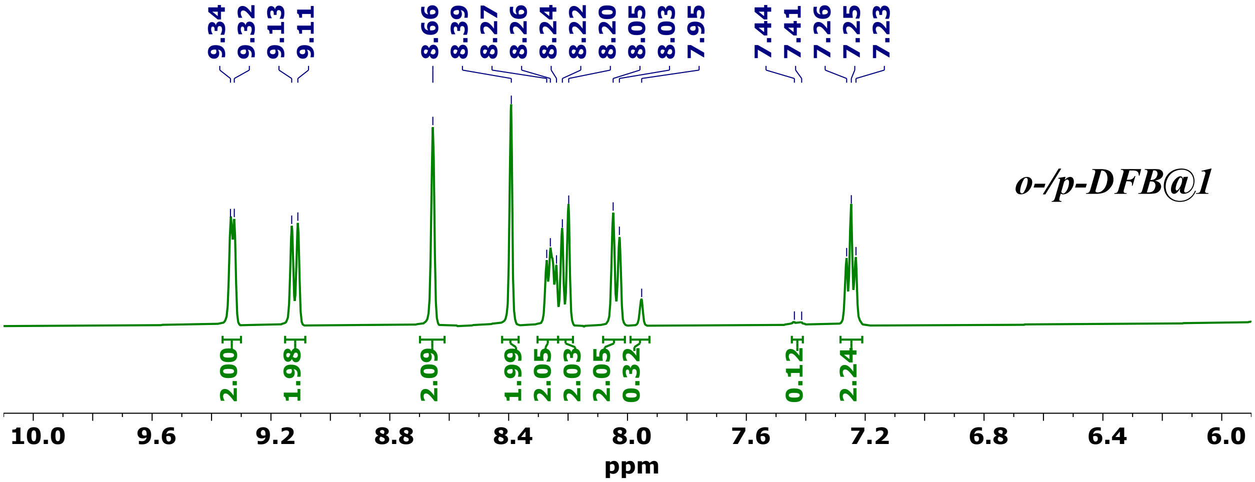

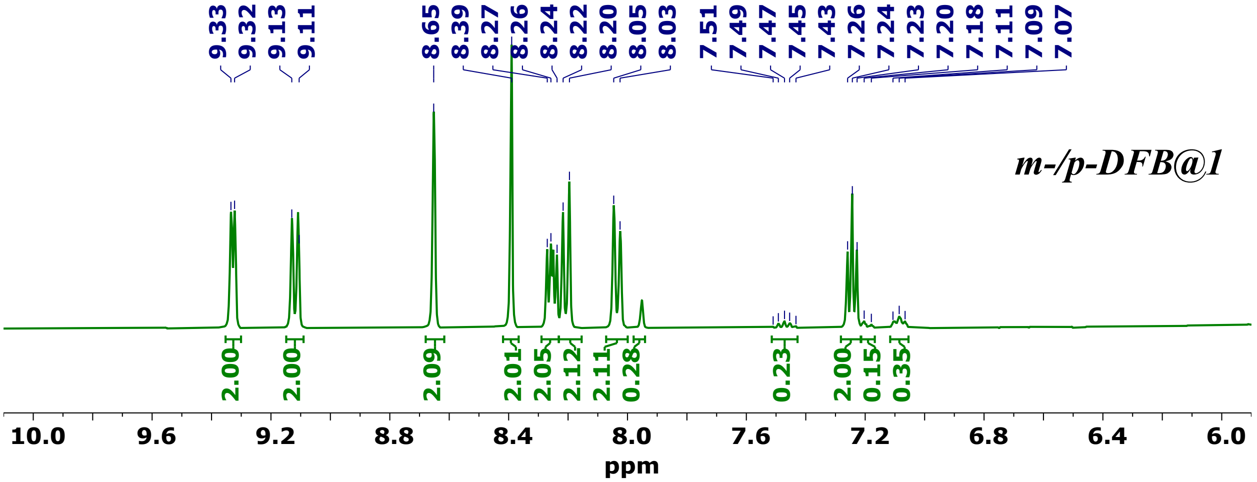

Fig. S8: Full range ^{1}H NMR spectra of digested crystals obtained from binary in-situ crystallization of difluorobenzenes.

NMR Spctra Showing PCP components along with halobenzene guests

NMR Spectra (Binary mixture of DCBs)

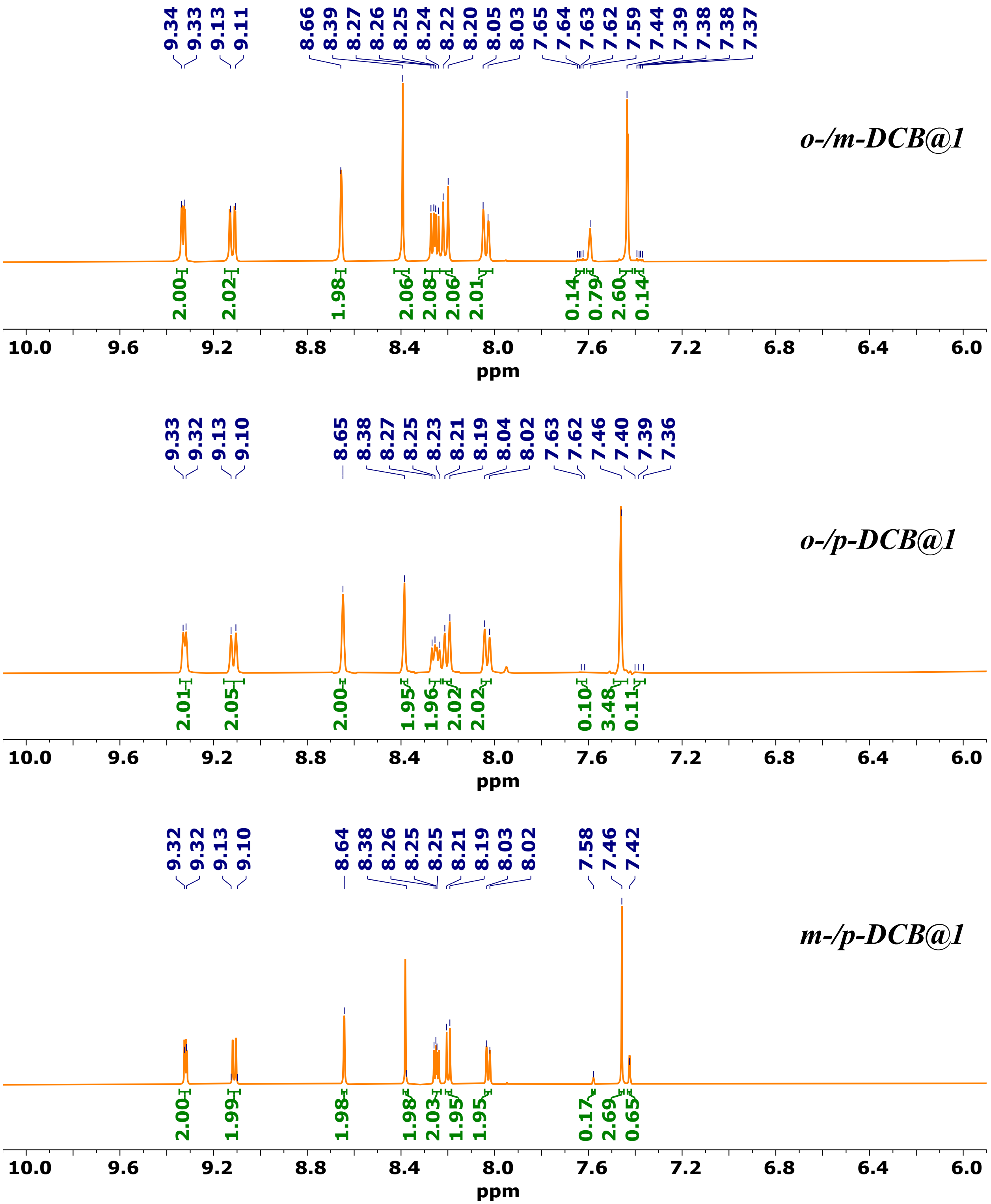

Fig. S9: Full range ^{1}H NMR spectra of digested crystals obtained from binary in-situ crystallization of dichlorobenzenes.

NMR Spctra Showing PCP components along with halobenzene guests

NMR Spectra (Binary mixture of DBBs)

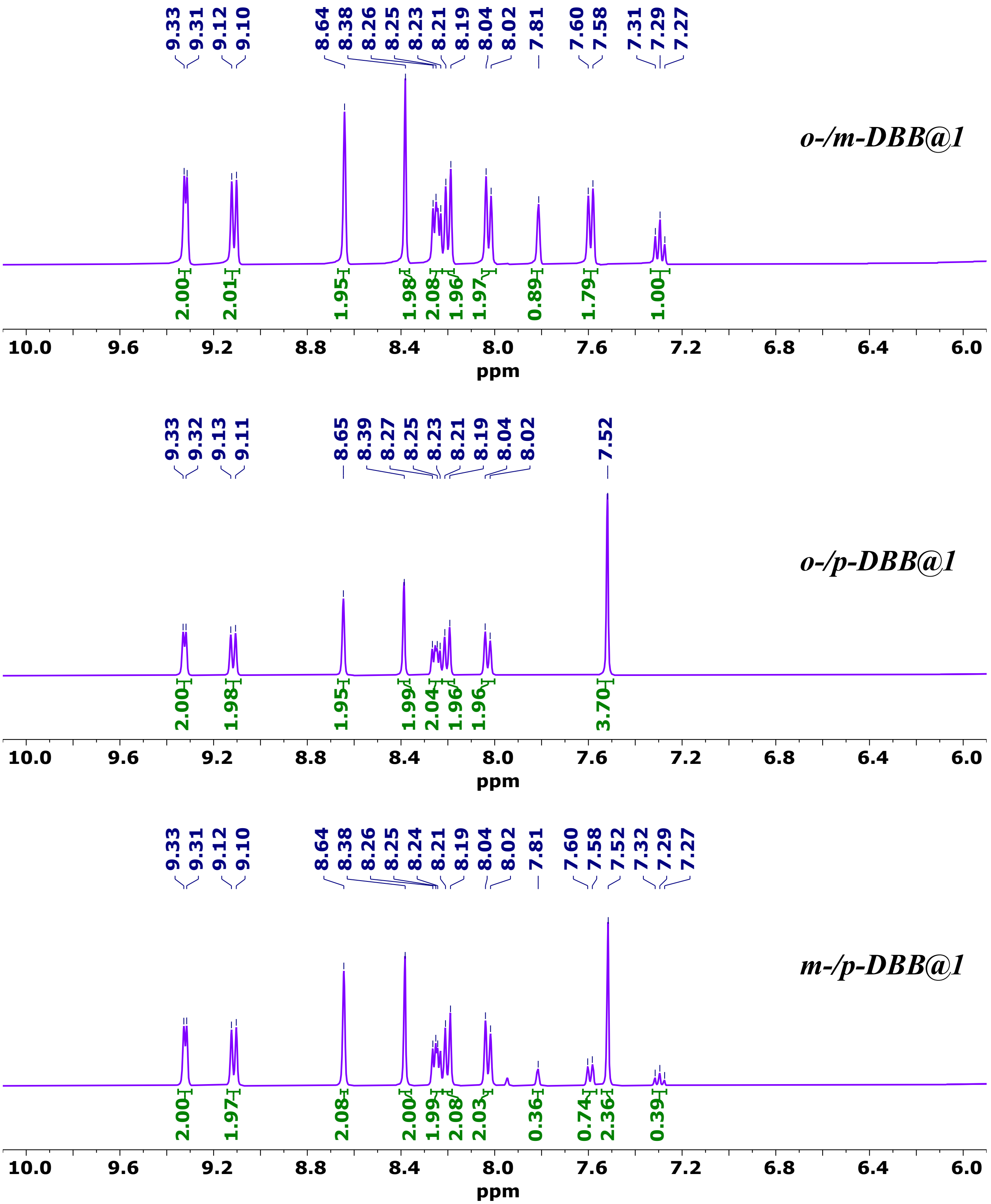

Fig. S10: Full range ^{1}H NMR spectra of digested crystals obtained from binary in-situ crystallization of dibromobenzenes.

NMR Spctra Showing PCP components along with halobenzene guests

NMR Spectra (Ternary mixture of DFBs, DCBs, DBBs)

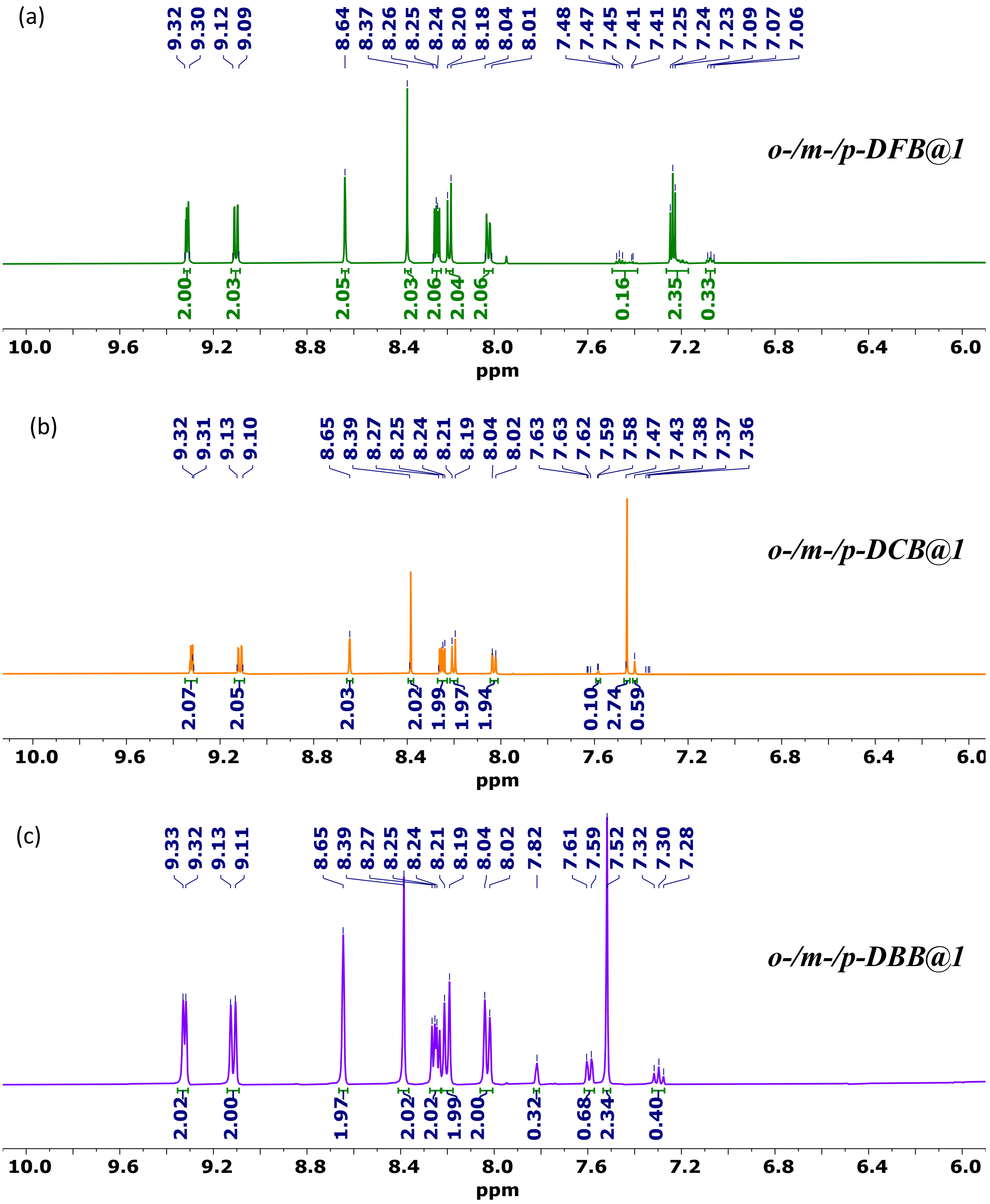

Fig. S11: Full range ^{1}H NMR spectra of digested crystals obtained from ternary in-situ crystallization of (a) difluorobenzenes, (b) dichlorobenzenes and (c) dibromobenzenes.

NMR Spctra Showing PCP components along with halobenzene guests

NMR Spectra (Binary mixture of FB, CB, BB)

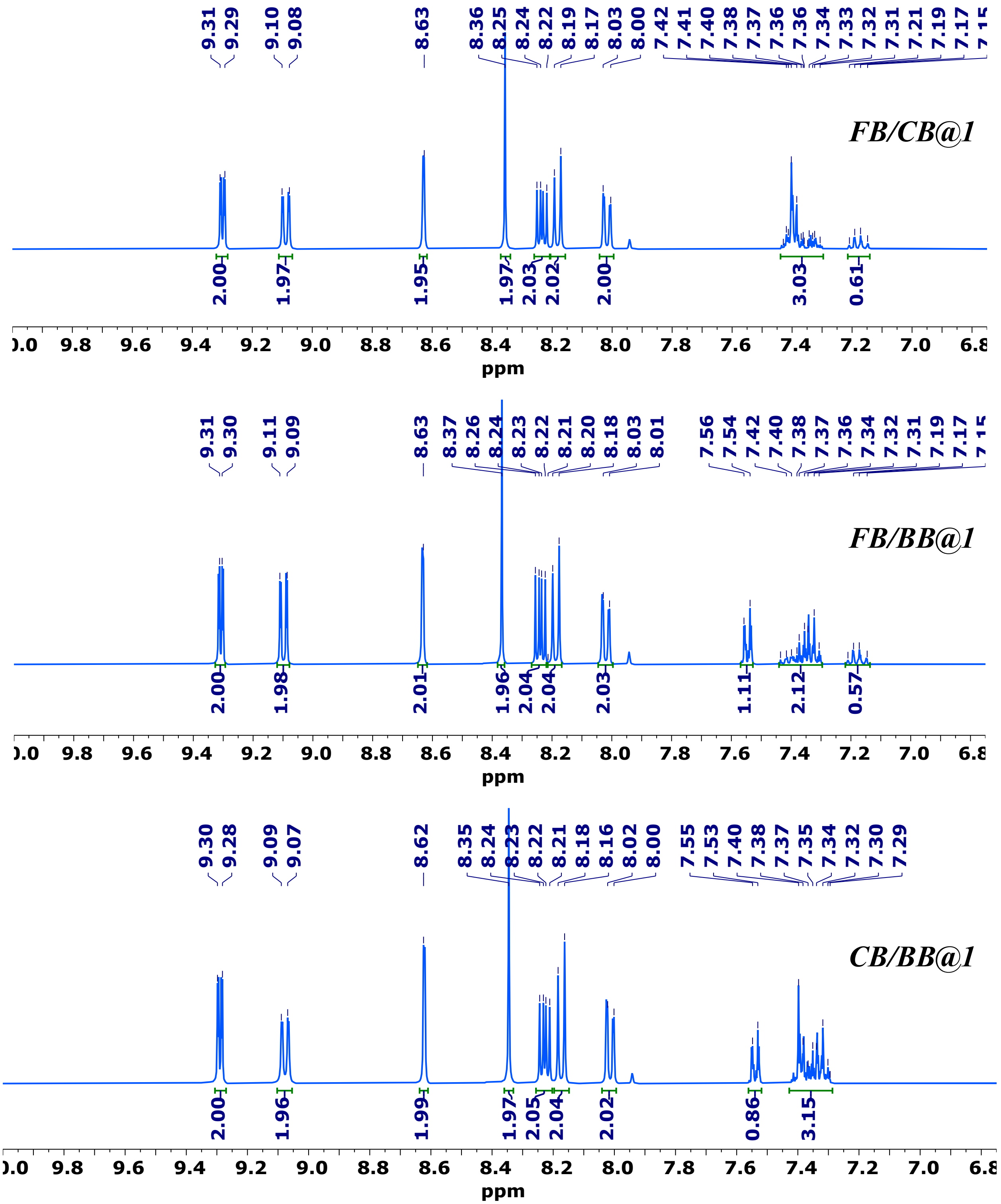

Fig. S12: Full range ^{1}H NMR spectra of digested crystals obtained from binary in-situ crystallization of monohalobenzenes.

NMR Spctra Showing PCP components along with halobenzene guests

NMR Spectra (Ternary mixture of FB, CB, BB)

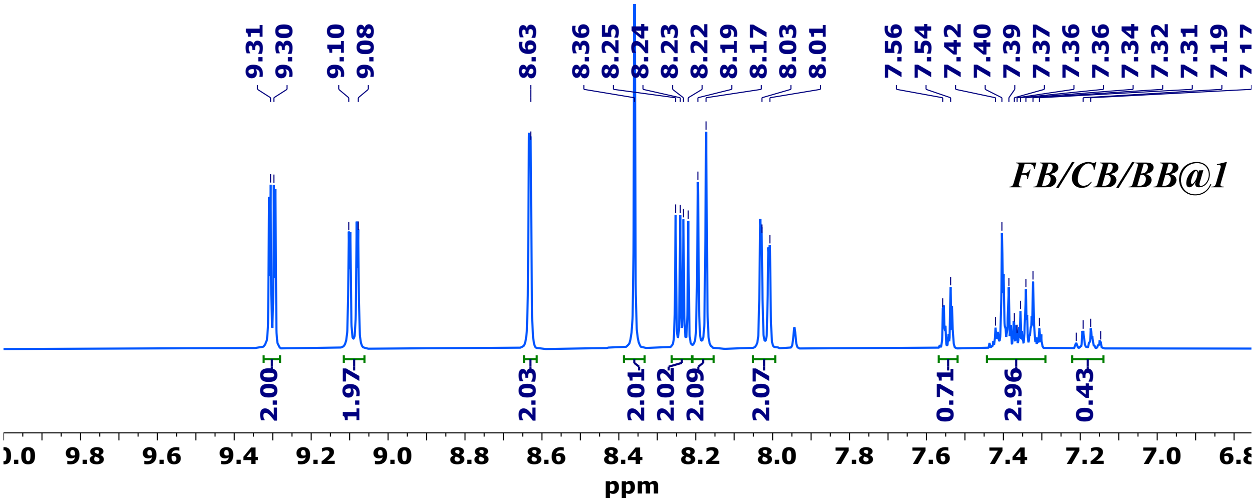

Fig. S13: Full range ^{1}H NMR spectra of digested crystals obtained from ternary in-situ crystallization of monohalobenzenes.

S5. (a) Binary Component Batch Reactions using Activated PCP

Procedure:

The As synthesized PCP [Zn(ndc)(o-phen)]·**DMF**)] was activated at 150 ^{o}C to remove the pore occupied DMF guests. 50 mg of activated PCP was added to an equimolar binary mixture of haloaromatics (dihalobenzenes) in hexane or dioxane. The batch reaction vial was closed and sealed using Teflon tape and kept at rest. After 48 h, the PCP was separated from the mixture and washed with hexane or dioxane to remove residual haloaromatics from the surface. It was then dried at room temperature overnight. For quantitative analysis of captured isomers within the activated PCP, a similar procedure has been followed as in **section S4** (**Figure S2**). The NMR analysis and selectivity values for the digested PCPs are shown below.

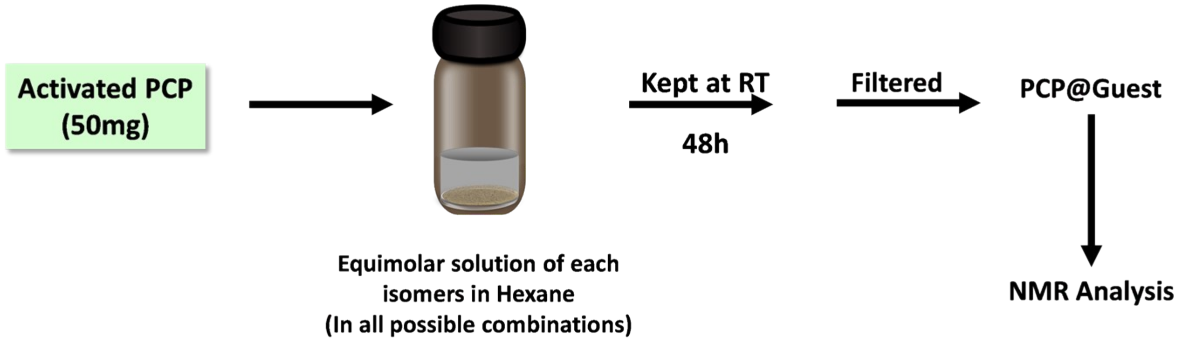

Fig. S14: Schematics for binary component batch reactions using activated PCP.

Table S4: Selectivity value for dihalobenzenes estimated from the NMR analyses of guest captured activated PCP, in binary component batch reactions.

	Mixture Components	Relative Concentrations
DFB	*o*-**DFB/** *m*-**DFB**	**14% / 86%**
	o-**DFB/** *p*-**DFB**	**11% / 89%**
	m-**DFB/** *p*-**DFB**	**24% / 76%**
DCB	*o*-**DCB/** *m*-**DCB**	**19% / 81%**
	o-**DCB/** *p*-**DCB**	**10% / 90%**
	m-**DCB/** *p*-**DCB**	**30% / 70%**
DBB	*o*-**DBB/** *m*-**DBB**	**0.001% / 99.9%**
	o-**DBB/** *p*-**DBB**	**0.02% / 99.8%**
	m-**DBB/** *p*-**DBB**	**47% / 53%**

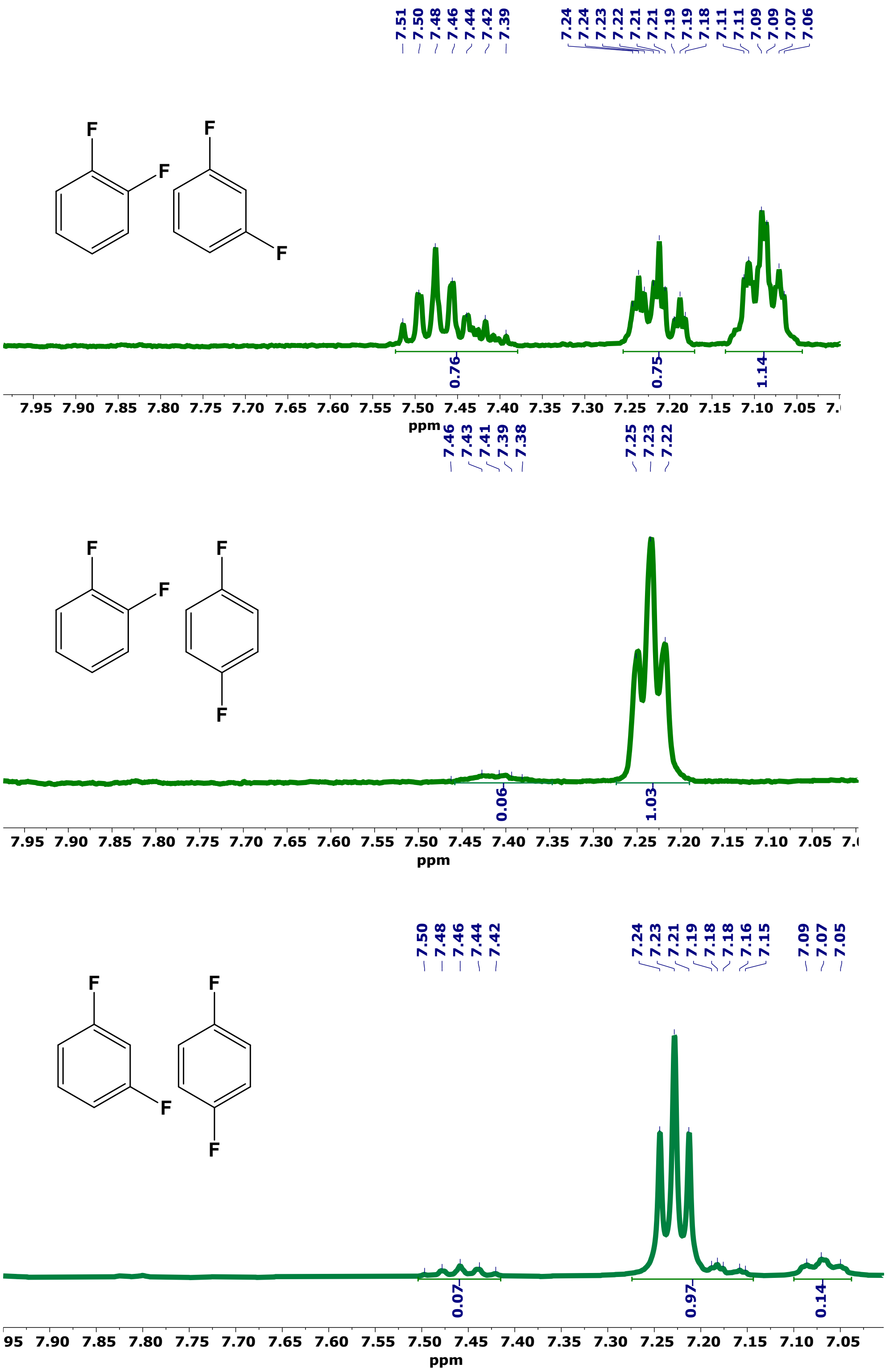

Fig. S15: ^{1}H NMR analyses of guest captured activated PCP, in binary component batch reactions for difluorobenzenes.

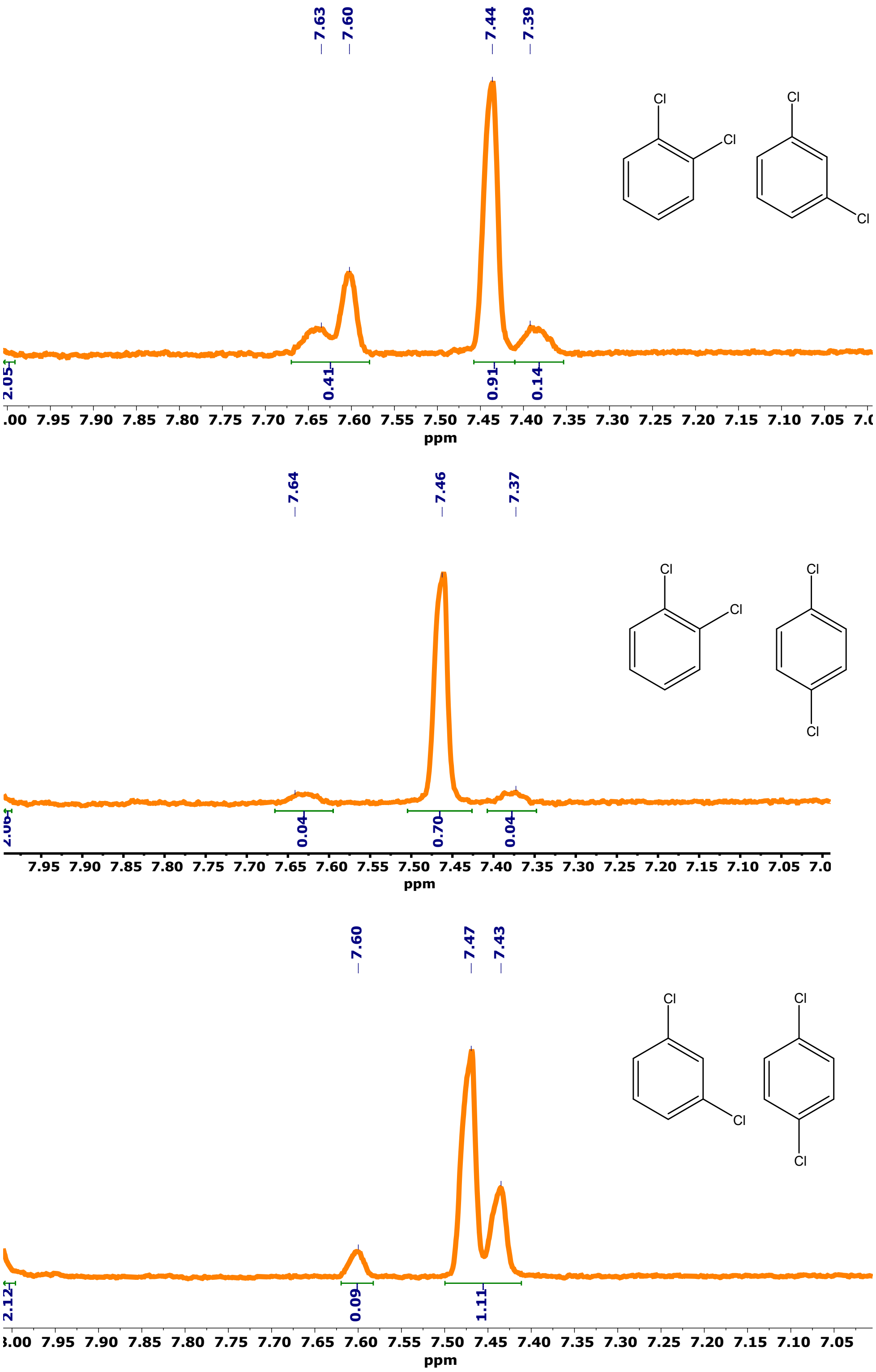

Fig. S16: ^{1}H NMR analyses of guest captured activated PCP, in binary component batch reactions for dichlorobenzenes.

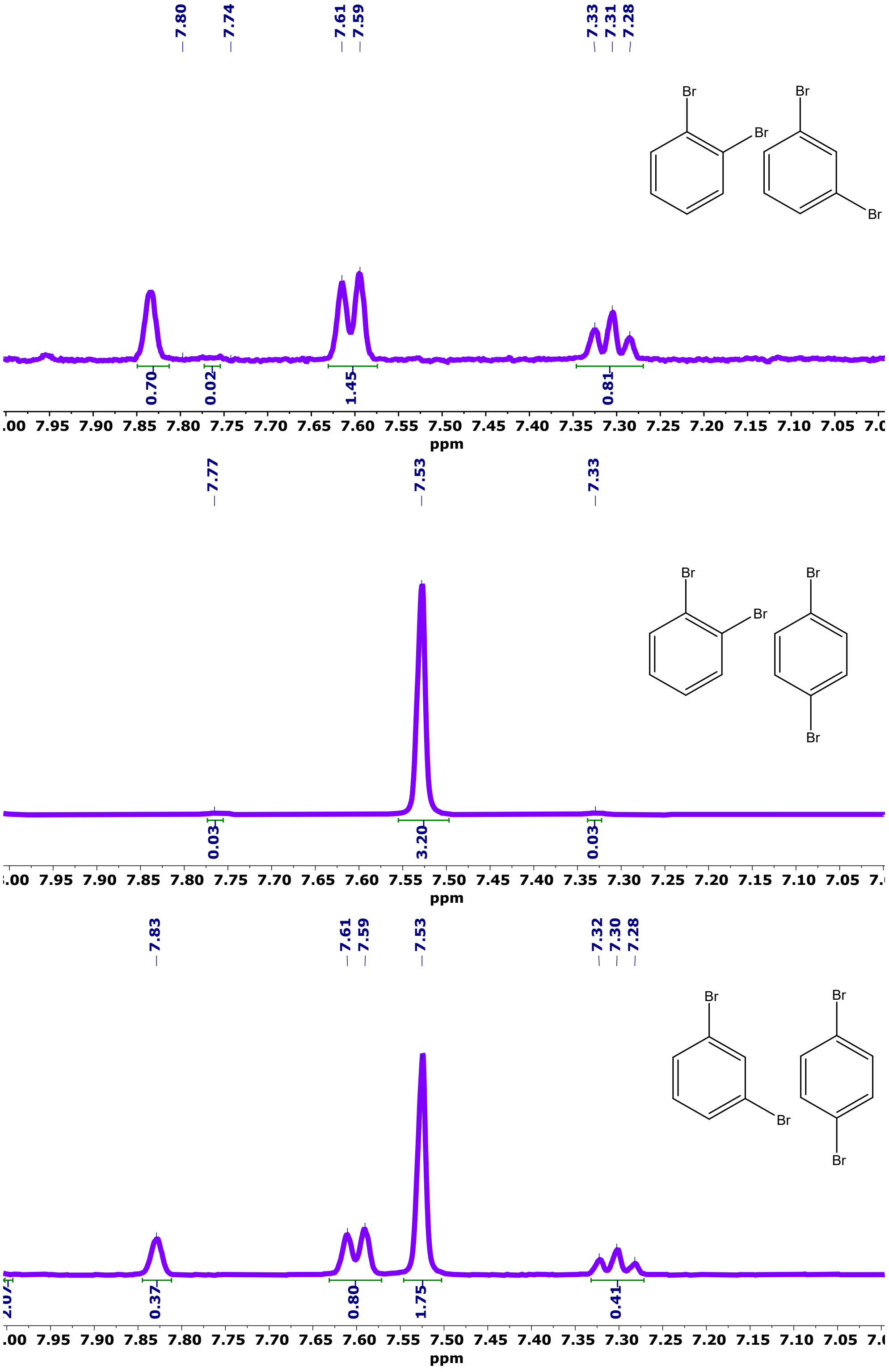

Fig. S17: ^{1}H NMR analyses of guest captured activated PCP, in binary component batch reactions for dibromobenzenes.

S5. (b) Trace Binary Component Batch Reactions using Activated PCP

Procedure: Similar procedure as S5(a) is followed except for the molar ratio of isomer mixtures. In the first case, a mixture of o-DBB and p-DBB in 0.8 to 0.2 molar ratio was considered respectively, i.e., 80% ortho isomer, 20% para isomer. Second, 0.9 to 0.1 binary molar ratio mixture of o-DBB and p-DBB was considered for batch reaction, i.e., 90% ortho isomer, 10% para isomer. The NMR analyses and relative concentrations of encapsulated isomers are shown below.

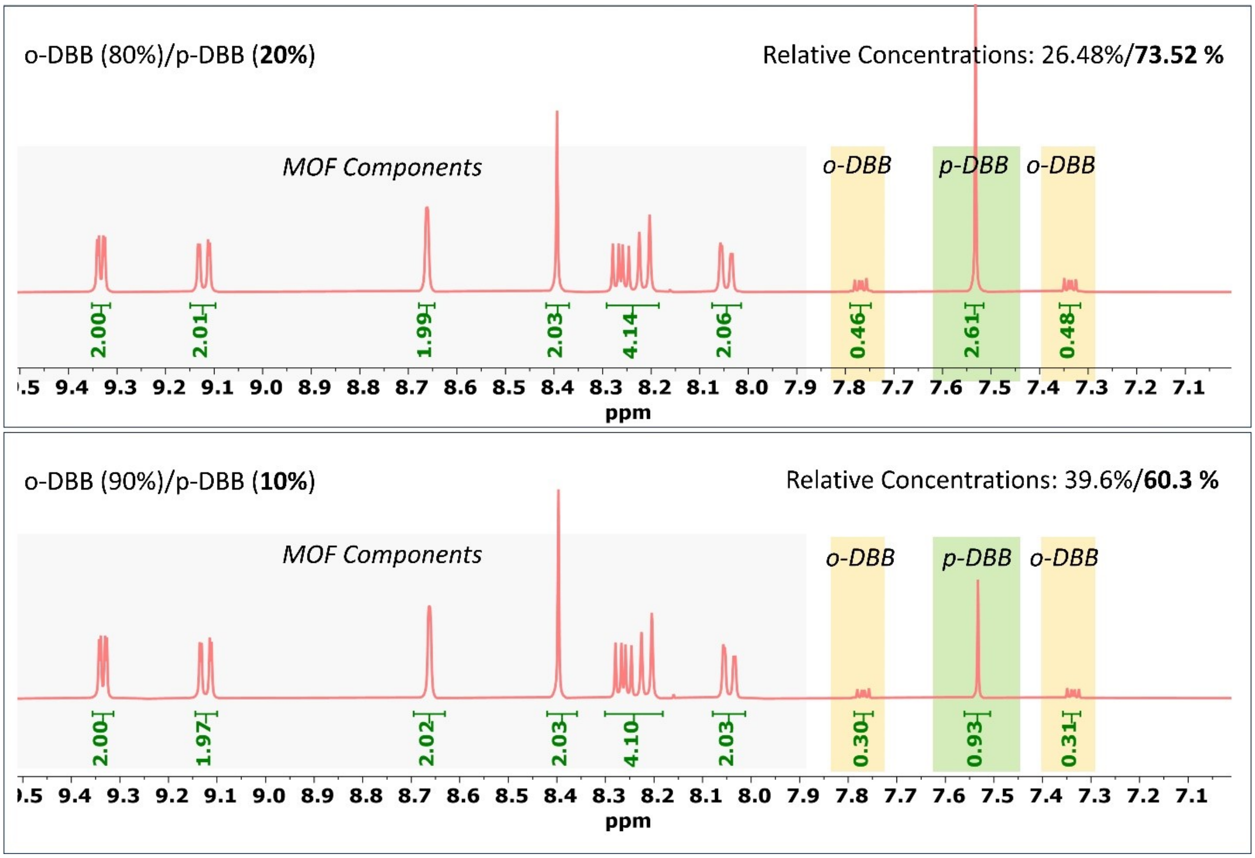

Fig. S18: ^{1}H NMR analyses and relative concentrations of isomer encapsulation by activated PCP from various trace concentrations of halobenzene mixtures.

S6. Single Crystal X-ray Diffraction measurements

Suitable single crystals of ***o-DFB@1, m-DFB@1, p-DFB@1, o-DCB@1, m-DCB@1, p-DCB@1, o-DBB@1, m-DBB@1, p-DBB@1, FB@1, CB@1, BB@1*** were mounted on a thin glass fiber. X-ray crystallographic data of such crystals were collected on a Bruker Smart-CCD diffractometer equipped with a normal focus, 2.4 kW sealed tube X-ray source with graphite monochromated Mo-Kα radiation (λ = 0.71073 Å) operating at 50 kV and 30 mA. The program SAINT[2] was used for integration of diffraction profiles, and absorption correction was made with SADABS[3] program. Structures were solved by SIR 92[4] and refined by the full-matrix least-squares method using SHELXL-97[5]. All hydrogen atoms were fixed in ideal positions by HFIX command. In addition, non-hydrogen atoms were refined anisotropically. All crystallographic and structure refinement data are summarized in Table **S5-S15**. All calculations were carried out using SHELXL-97, PLATON[6], SHELXS-97[7] and X-Seed Ver 4[8]. The crystal structures can be obtained from CCDC number **2266788-2266798**. [Note: The structure of *o-DBB@1* could not be refined, due to highly disordered guest molecule (even at 100K) and the structure was modelled analogous to *o-DCB@1*]

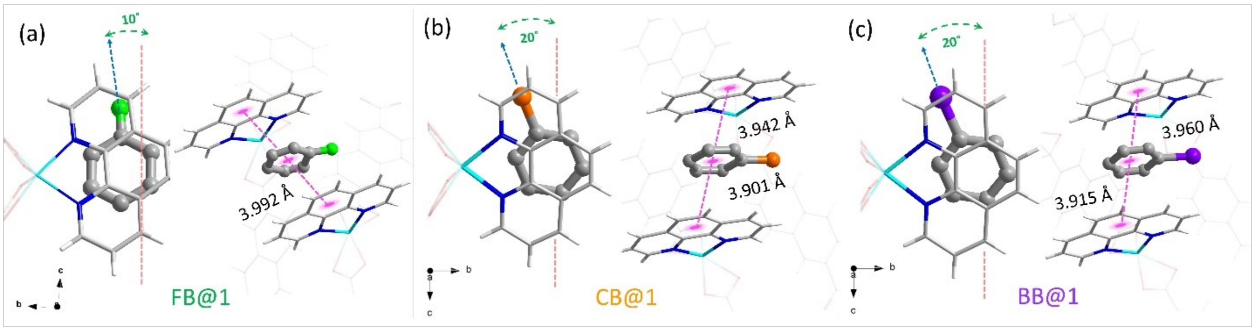

Fig. S19: Crystal Structure of monohalobenzene encapsulated PCP (1): FB@1, CB@1 and BB@1

Table S5

***o-DFB@1* (o-difluorobenzene encapsulated PCP crystals)**

**Empirical formula**	$C_{30}H_{18}N_2O_4F_2Zn$
**Formula weight**	573.85
**Crystal system**	monoclinic
**Space group**	P2/n
a **(Å)**	14.980 (3)
b **(Å)**	9.8498 (17)
c **(Å)**	16.639 (3)
α **(deg)**	90
β **(deg)**	94.860 (7)
γ **(deg)**	90
V **(Å^3)**	2446.3 (8)
Z	4
T **(K)**	100
**D_c (g cm^{-3})**	1.558
**μ (mm^{-1})**	1.061
F **(000)**	1168
**ϑ_{max} (deg)**	25.0
**λ (Mo Kα)**	0.71073
**Total data**	29522
**Unique data, R_{int}**	4301, 0.111
**Data [I >2σ(I)]**	3002
**R^a**	0.0778
**R_w^b**	0.2122
**GOF**	1.04

$^aR = \sum||F_o| - |F_c||/\sum|F_o|$, $^bR_w = [\sum\{w(F_o{}^2 - F_c{}^2)^2\}/\sum\{w(F_o{}^2)^2\}]^{1/2}$.

Table S6

m-DFB@1 **(m-difluorobenzene encapsulated PCP crystals)**

**Empirical formula**	$C_{30}H_{18}N_2O_4F_2Zn$
**Formula weight**	573.85
**Crystal system**	monoclinic
**Space group**	P2/c
a **(Å)**	7.8066 (1)
b **(Å)**	9.8765 (2)
c **(Å)**	16.3671 (3)
α **(deg)**	90
β **(deg)**	92.361 (1)
γ **(deg)**	90
V **(Å^3)**	1260.86 (4)
Z	2
T **(K)**	293
D_c **(g cm^{-3})**	1.512
μ **(mm^{-1})**	1.029
F **(000)**	584
ϑ_{max} **(deg)**	28.3
λ **(Mo Kα)**	0.71073
**Total data**	28012
**Unique data, R_{int}**	3105, 0.028
**Data [*I* >2σ(I)]**	2915
**R[a]**	0.028
**R_w[b]**	0.0799
**GOF**	1.07

[a]R = $\sum||F_o| - |F_c||/\sum|F_o|$, [b]$R_w = [\sum\{w(F_o^2 - F_c^2)^2\}/\sum\{w(F_o^2)^2\}]^{1/2}$.

Table S7

p-DFB@1 **(*p*-difluorobenzene encapsulated PCP crystals)**

**Empirical formula**	$C_{30}H_{18}N_2O_4F_2Zn$
**Formula weight**	573.85
**Crystal system**	monoclinic
**Space group**	P2/c
a **(Å)**	7.8873 (3)
b **(Å)**	9.9771 (4)
c **(Å)**	16.0181 (7)
α **(deg)**	90
β **(deg)**	93.433 (1)
γ **(deg)**	90
**V (Å^3)**	1258.24 (9)
Z	2
T **(K)**	293
**D_c (g cm^{-3})**	1.515
**μ (mm^{-1})**	1.031
F **(000)**	584
**ϑ_{max} (deg)**	28.3
λ **(Mo Kα)**	0.71073
**Total data**	27356
**Unique data, R_{int}**	3145, 0.137
**Data [*I* >2σ(I)]**	1924
**R^a**	0.0461
**R_w^b**	0.1133
**GOF**	1.01

$^aR = \sum||F_o| - |F_c||/\sum|F_o|$, $^bR_w = [\sum\{w(F_o{}^2 - F_c{}^2)^2\}/\sum\{w(F_o{}^2)^2\}]^{1/2}$.

Table S8

o-DCB@1 **(*o*-dichlorobenzene encapsulated PCP crystals)**

**Empirical formula**	$C_{30}H_{17}N_2O_4Cl_2Zn$
**Formula weight**	605.75
**Crystal system**	orthorhombic
**Space group**	pbcn
a **(Å)**	7.5165 (2)
b **(Å)**	21.9107 (6)
c **(Å)**	15.9258 (4)
α **(deg)**	90
β **(deg)**	90
γ **(deg)**	90
V **($Å^3$)**	2622.85 (12)
Z	4
T **(K)**	293
**D_c (g cm^{-3})**	1.534
**μ (mm^{-1})**	1.181
F **(000)**	1228
**ϑ_{max} (deg)**	25.0
λ **(Mo Kα)**	0.71073
**Total data**	154088
**Unique data, R_{int}**	2301, 0.065
**Data [*I* >2σ(I)]**	2034
**R^a**	0.0988
**R_w^b**	0.3735
**GOF**	1.82

$^a R = \sum\|F_o| - |F_c\|/\sum|F_o|$, $^b R_w = [\sum\{w(F_o{}^2 - F_c{}^2)^2\}/\sum\{w(F_o{}^2)^2\}]^{1/2}$.

Table S9

m-DCB@1 **(*m*-dichlorobenzene encapsulated PCP crystals)**

**Empirical formula**	$C_{30}H_{18}N_2O_4Cl_2Zn$
**Formula weight**	606.75
**Crystal system**	Monoclinic
**Space group**	P2/c
a **(Å)**	7.5498 (3)
b **(Å)**	9.7529 (3)
c **(Å)**	17.1813 (6)
α **(deg)**	90
β **(deg)**	91.296 (1)
γ **(deg)**	90
V **(Å^3)**	1264.91 (8)
Z	2
T **(K)**	293
**D_c (g cm^{-3})**	1.593
**μ (mm^{-1})**	1.224
F **(000)**	616
**ϑ_{max} (deg)**	32.1
λ **(Mo Kα)**	0.71073
**Total data**	31726
**Unique data, R_{int}**	4397, 0.15
**Data [*I* >2σ(I)]**	2097
**R[a]**	0.0534
**R_w[b]**	0.1093
**GOF**	0.99

[a]$R = \sum||F_o| - |F_c||/\sum|F_o|$, [b]$R_w = [\sum\{w(F_o{}^2 - F_c{}^2)^2\}/\sum\{w(F_o{}^2)^2\}]^{1/2}$.

Table S10

p-DCB@1 **(*p*-dichlorobenzene encapsulated PCP crystals)**

**Empirical formula**	$C_{30}H_{18}N_2O_4Cl_2Zn$
**Formula weight**	606.75
**Crystal system**	Monoclinic
**Space group**	P2/c
a **(Å)**	7.6348 (1)
b **(Å)**	9.8825 (2)
c **(Å)**	17.0598 (4)
α **(deg)**	90
β **(deg)**	90.083 (1)
γ **(deg)**	90
V **($Å^3$)**	1287.18 (4)
Z	2
T **(K)**	293
**D_c (g cm^{-3})**	1.566
**μ (mm^{-1})**	1.206
F **(000)**	616
**ϑ_{max} (deg)**	28.3
λ **(Mo Kα)**	0.71073
**Total data**	28031
**Unique data, R_{int}**	3219, 0.045
**Data [*I* >2σ(I)]**	2787
**R^a**	0.0349
**$R_w{}^b$**	0.1029
**GOF**	1.05

$^aR = \sum||F_o| - |F_c||/\sum|F_o|$, $^bR_w = [\sum\{w(F_o{}^2 - F_c{}^2)^2\}/\sum\{w(F_o{}^2)^2\}]^{1/2}$.

Table S11

m-DBB@1 **(*m*-dibromobenzene encapsulated PCP crystals)**

**Empirical formula**	$C_{30}H_{18}N_2O_4Br_2Zn$
**Formula weight**	695.65
**Crystal system**	Monoclinic
**Space group**	P2/c
a **(Å)**	7.5558 (2)
b **(Å)**	9.7064 (2)
c **(Å)**	17.3881 (4)
α **(deg)**	90
β **(deg)**	92.212 (1)
γ **(deg)**	90
V **(Å^3)**	1274.29 (5)
Z	2
T **(K)**	293
**D_c (g cm^{-3})**	1.813
**μ (mm^{-1})**	4.141
F **(000)**	688
**ϑ_{max} (deg)**	28.3
λ **(Mo Kα)**	0.71073
**Total data**	83765
**Unique data, R_{int}**	3158, 0.035
**Data [*I* >2σ(I)]**	2928
**R^a**	0.0441
**R_w^b**	0.1435
**GOF**	1.11

$^a R = \sum||F_o| - |F_c||/\sum|F_o|$, $^b R_w = [\sum\{w(F_o{}^2 - F_c{}^2)^2\}/\sum\{w(F_o{}^2)^2\}]^{1/2}$.

Table S12

p-DBB@1 **(*p*-dibromobenzene encapsulated PCP crystals)**

**Empirical formula**	$C_{30}H_{18}N_2O_4Br_2Zn$
**Formula weight**	695.65
**Crystal system**	Monoclinic
**Space group**	P2/c
a **(Å)**	7.6210 (1)
b **(Å)**	9.8308 (2)
c **(Å)**	17.4204 (4)
α **(deg)**	90
β **(deg)**	91.107 (1)
γ **(deg)**	90
V **($Å^3$)**	1304.90 (5)
Z	2
T **(K)**	293
**D_c (g cm^{-3})**	1.770
**μ (mm^{-1})**	4.044
F **(000)**	688
**ϑ_{max} (deg)**	28.3
**λ (Mo Kα)**	0.71073
**Total data**	28081
**Unique data, R_{int}**	3247, 0.048
**Data [*I* >2σ(I)]**	2564
**R^a**	0.0507
**R_w^b**	0.1323
**GOF**	1.05

$^aR = \sum||F_o| - |F_c||/\sum|F_o|$, $^bR_w = [\sum\{w(F_o{}^2 - F_c{}^2)^2\}/\sum\{w(F_o{}^2)^2\}]^{1/2}$.

Table S13

***FB@1* (Fluorobenzene encapsulated PCP crystals)**

**Empirical formula**	$C_{30}H_{19}N_2O_4FZn$
**Formula weight**	555.86
**Crystal system**	Monoclinic
**Space group**	$P2_1/c$
***a* (Å)**	7.6189 (2)
***b* (Å)**	19.5052 (6)
***c* (Å)**	16.4250 (5)
***α* (deg)**	90
***β* (deg)**	91.019 (1)
***γ* (deg)**	90
***V* (Å^3)**	2440.50 (12)
Z	4
***T* (K)**	100
**D_c (g cm^{-3})**	1.513
**μ (mm^{-1})**	1.055
***F* (000)**	1136
**ϑ_{max} (deg)**	26.4
**λ (Mo Kα)**	0.71073
**Total data**	153790
**Unique data, R_{int}**	5008, 0.060
**Data [*I* >2σ(I)]**	4697
**R^a**	0.0525
**R_w^b**	0.1432
**GOF**	1.15

$^aR = \sum||F_o| - |F_c||/\sum|F_o|$, $^bR_w = [\sum\{w(F_o{}^2 - F_c{}^2)^2\}/\sum\{w(F_o{}^2)^2\}]^{1/2}$.

Table S14

***CB@1* (chlorobenzene encapsulated PCP crystals)**

**Empirical formula**	$C_{30}H_{19}N_2O_4ClZn$
**Formula weight**	572.31
**Crystal system**	Monoclinic
**Space group**	$P2_1/c$
a **(Å)**	7.774 (2)
b **(Å)**	19.6561 (5)
c **(Å)**	16.5227 (4)
α **(deg)**	90
β **(deg)**	91.553 (1)
γ **(deg)**	90
V **(Å^3)**	2524.95 (11)
Z	4
T **(K)**	293
**D_c (g cm^{-3})**	1.505
**μ (mm^{-1})**	1.119
F **(000)**	1168
**ϑ_{max} (deg)**	28.3
λ **(Mo Kα)**	0.71073
**Total data**	97949
**Unique data, R_{int}**	6253, 0.039
**Data [*I* >2σ(I)]**	5548
**R^a**	0.0521
**R_w^b**	0.1622
**GOF**	1.08

$^a R = \sum||F_o| - |F_c||/\sum|F_o|$, $^b R_w = [\sum\{w(F_o{}^2 - F_c{}^2)^2\}/\sum\{w(F_o{}^2)^2\}]^{1/2}$.

Table S15

***BB@1* (bromobenzene encapsulated PCP crystals)**

**Empirical formula**	$C_{30}H_{19}N_2O_4BrZn$
**Formula weight**	616.76
**Crystal system**	Monoclinic
**Space group**	$P2_1/c$
***a* (Å)**	7.7941 (7)
***b* (Å)**	19.4730 (18)
***c* (Å)**	16.6246 (12)
***α* (deg)**	90
***β* (deg)**	91.402 (1)
***γ* (deg)**	90
**V (Å^3)**	25222.4 (4)
Z	4
***T* (K)**	293
**D_c (g cm^{-3})**	1.624
**μ (mm^{-1})**	2.598
***F* (000)**	1240
**ϑ_{max} (deg)**	28.4
**λ (Mo Kα)**	0.71073
**Total data**	53254
**Unique data, R_{int}**	6219, 0.121
**Data [I >2σ(I)]**	4068
**R^a**	0.0848
**R_w^b**	0.2387
**GOF**	1.04

$^aR = \sum||F_o| - |F_c||/\sum|F_o|$, $^bR_w = [\sum\{w(F_o{}^2 - F_c{}^2)^2\}/\sum\{w(F_o{}^2)^2\}]^{1/2}$.

S7. DSC Analysis

Procedure: The obtained single crystals (2-5mg) of individual halobenzene guest encapsulated PCP were kept in aluminum pans and clipped with aluminum lids (DSC cells) at moderately high pressure to ensure contact between sample and top lid. Controlled thermal treatment was programmed at 5 ^{0}C/min in inert atmosphere (N_2). The heat flow was measured and respective enthalpies were estimated using TA Universal analysis software. The enthalpy values are tabulated in **Table S16**. The peak area in each plot is integrated 5 times. Further, the highest and lowest values were considered for getting the mean value along with error. The instrument generated data after enthalpy calculations are also mentioned in **Fig. S21-S24**.

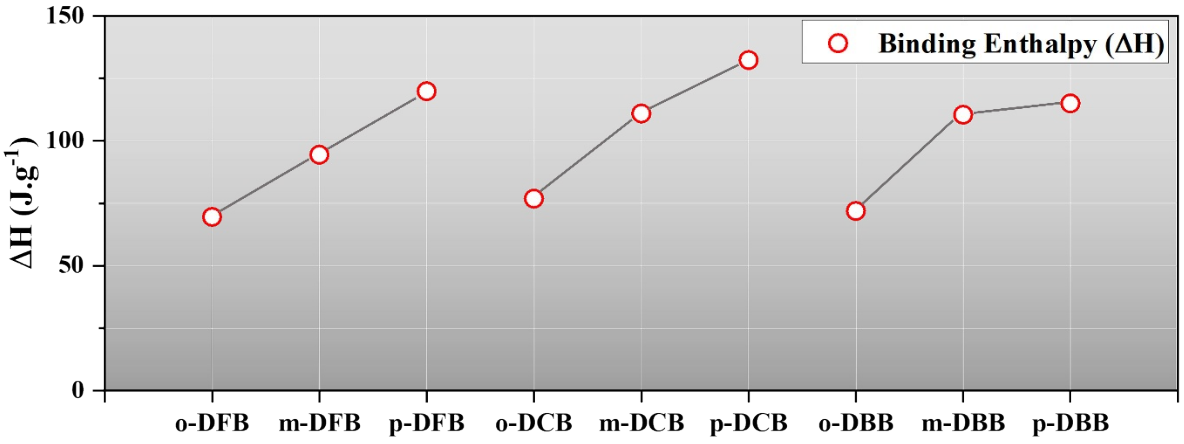

Fig. S20: Comparison of enthalpies of binding of the dihalobenzenes (**Guest@1**).

Table S16: Binding enthalpy of Guest@1 crystals measured by differential scanning calorimetry experiments.

**Samples**	**Enthalpy of Guest release ($J.g^{-1}$)**
o-***DFB@1***	69.53 ± 4.39
m-***DFB@1***	94.375 ± 1.825
p-***DFB@1***	119.85 ± 5.05
o-***DCB@1***	76.86 ± 2.28
m-***DCB@1***	110.9 ± 1.9
p-***DCB@1***	132.2 ± 4.1
o-***DBB@1***	71.92 ± 1.14
m-***DBB@1***	109.95 ± 2.05
p-***DBB@1***	114.95 ± 2.85
FB*@1*	39.555 ± 1.815
CB*@1*	103.65 ± 2.35
BB*@1*	116.9 ± 4.5

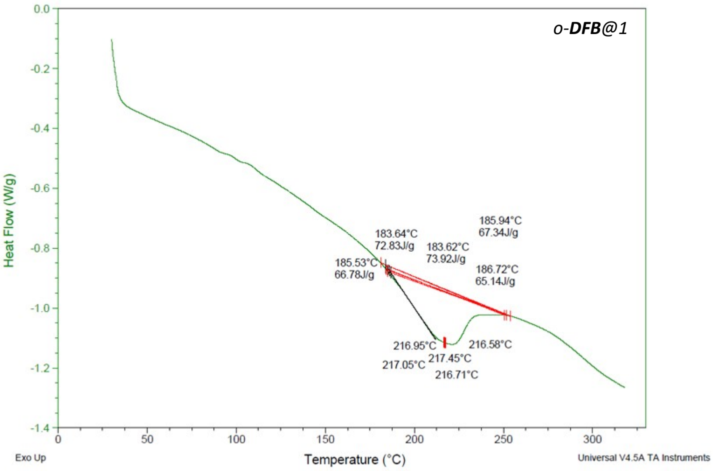

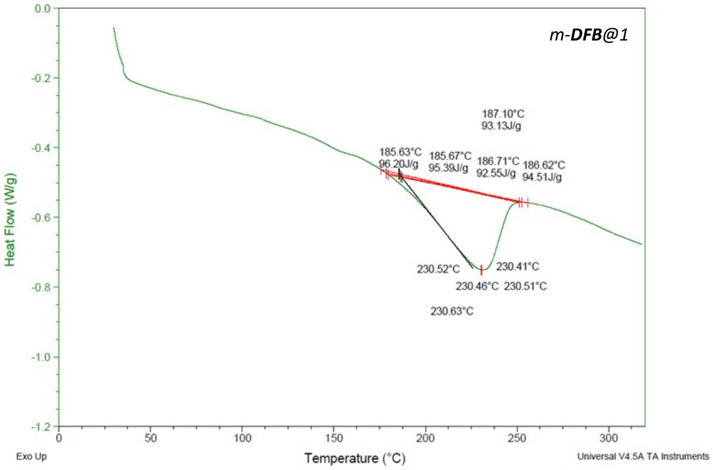

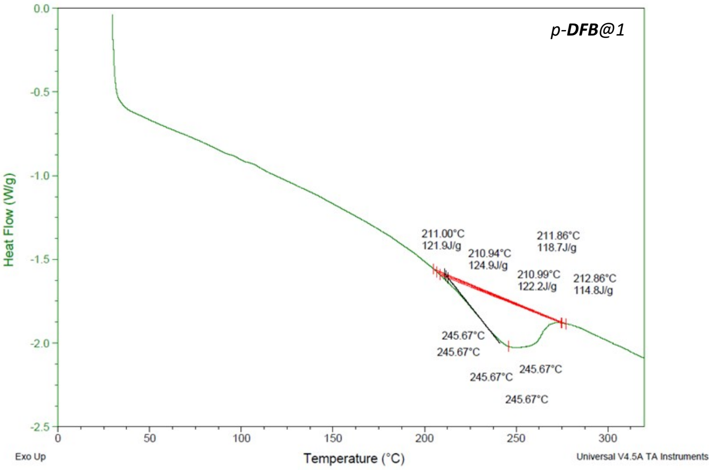

Fig. S21: DSC plots of (a) o-DFB@1, (b) m-DFB@1, and (c) p-DFB@1.

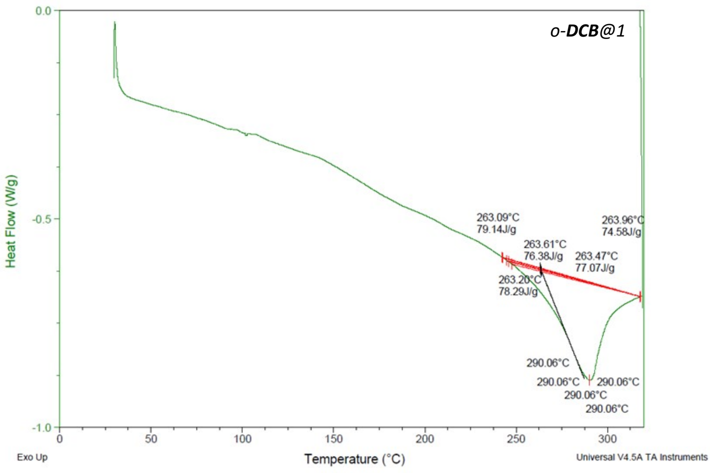

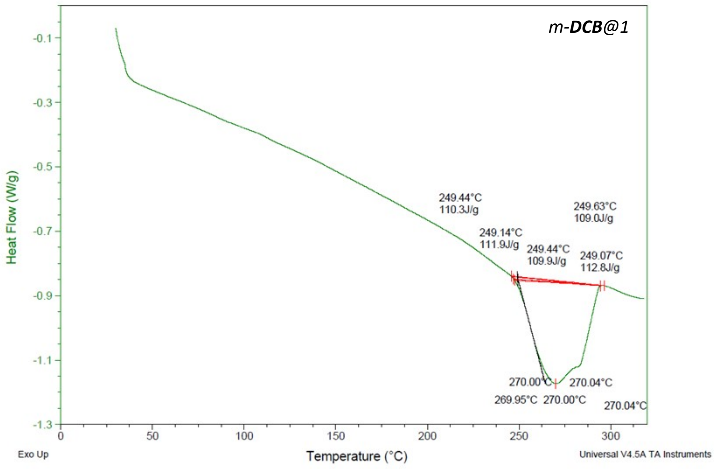

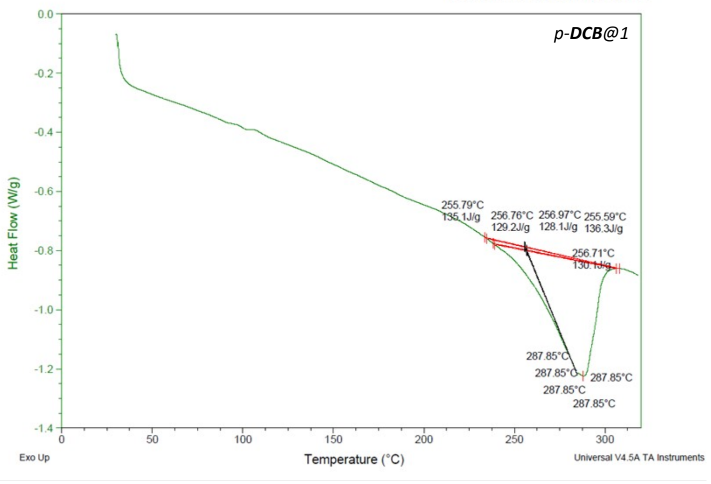

Fig. S22: DSC plots of (a) o-DCB@1, (b) m-DCB@1, and (c) p-DCB@1.

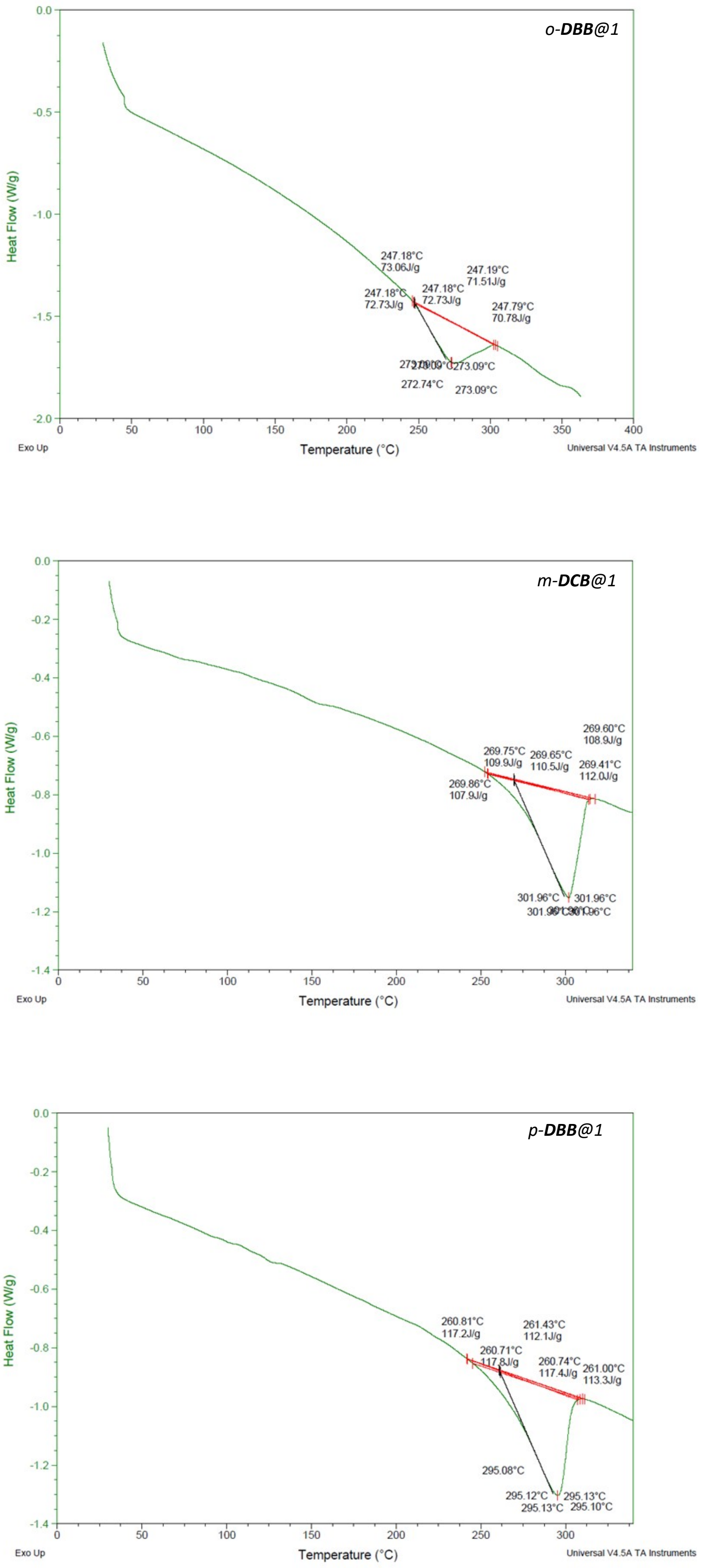

Fig. S23: DSC plots of (a) o-DBB@1, (b) m-DBB@1, and (c) p-DBB@1.

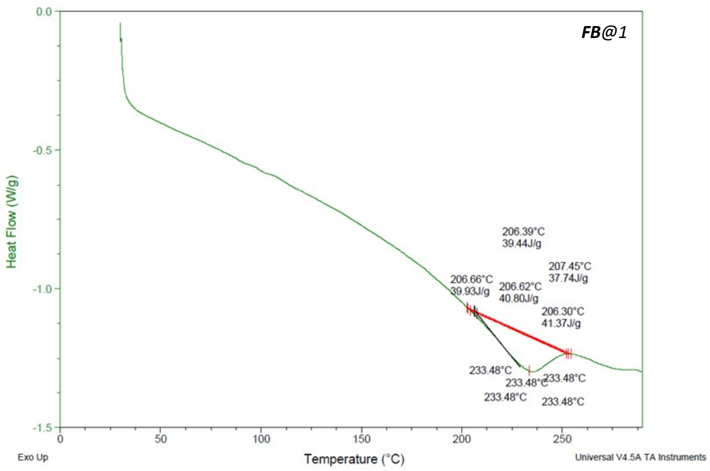

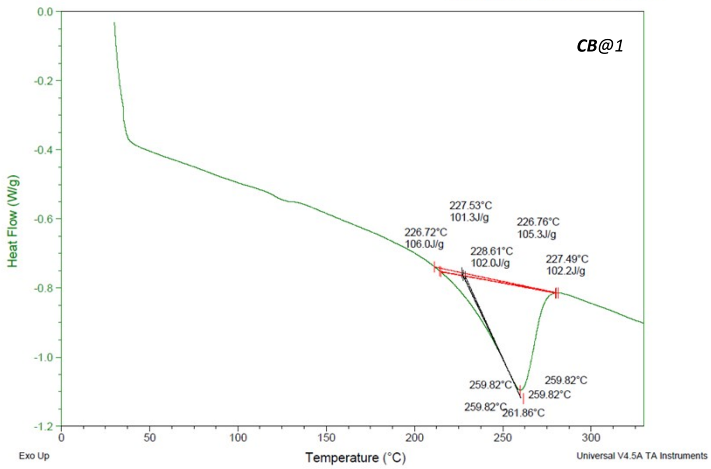

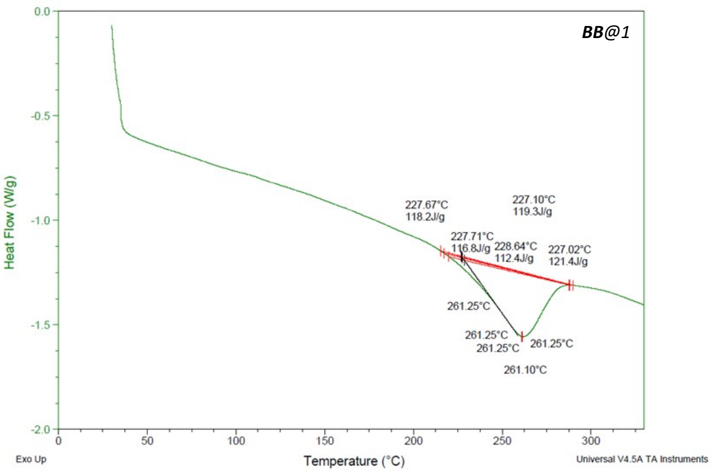

Fig. S24: DSC plots of (a) FB@1, (b) CB@1, and (c) BB@1.

S8. Computational Details

S8a. *Ab Initio* Cell Optimizations

Density functional theory (DFT) based calculations for PCPs were performed using the Gaussian Plane Wave (GPW)[9] method implemented in the Quickstep[10,11] module of the CP2K-7.1[11,12] package. Perdew-Burke-Ernzerhof (PBE)[13] exchange-correlation (XC) functional with Grimme's D3[14,15] dispersion corrections were used. Core-electrons for all the atoms were represented using the Goedecker-Teter-Hutter (GTH)[16–18] pseudopotentials while Triple-ζ with two sets of polarization function basis sets optimized for molecular calculations (TZV2P-MOLOPT-GTH)[18] was used to represent the valence electrons of hydrogen, nitrogen, carbon, oxygen, fluorine, chlorine, and bromine atoms, while short-range variants (TZV2P-MOLOPT-SR-GTH) were used for zinc and iodine atoms. Gaussian orbitals were mapped onto five multi-grids in all the GPW calculations. Plane-wave and relative cut-offs were set to 400 and 40 Ry, respectively. Convergence criteria for both inner and outer self-consistent field (SCF) cycles were set to 1.0×10^{-7} Ha. Poisson solver[19–22] was employed to calculate the electronic contribution to Hamiltonian.

DFT-based cell optimizations (CO) of supercells of all the experimentally determined crystal structures of PCPs were carried out. The required stress tensor was calculated using the analytical method implemented in CP2K, and root mean square (RMS) and maximum force convergence were set to 3.0×10^{-4} and 4.5×10^{-4} $Ha{\cdot}a_0^{-1}$, respectively. Further, no restriction was imposed on changes in the cell lengths and angles of the PCP supercells during the cell optimizations. Default values were used for all other DFT and optimization parameters. For the calculations, experimentally determined unit cells were replicated such that the supercell volume is ca. 5000 $Å^3$ and any edge of a given supercell is at least 15 Å in length (**Table S17**). As a result, each supercell constructed contains eight guest molecules.

S8b. Binding Energy Calculations

For a given configuration of guest molecules in the framework, the energies of guest (E_{guest}), framework (E_{PCP}), and combined framework-guest ($E_{PCP+guest}$) systems are calculated separately using DFT single-point energy calculations. The binding energy per guest molecule (ΔE) is given by,

$$\Delta E = \frac{E_{PCP+guest} - (E_{PCP} + E_{guest})}{n}$$

where, 'n' is the number of guest molecules.

S8c. Electron Density Difference Map Calculations

Electron density difference maps can be used as a visual aid to understand what regions of the framework interact with a guest molecule. Analogous to the binding energy, the electron density difference ($\Delta\rho(\vec{r})$) can be calculated using the electron density of the framework and guest system $(\rho_{PCP\,+\,guest}(\vec{r}))$, electron density of framework $(\rho_{PCP}(\vec{r}))$, and electron density of guest molecules $(\rho_{guest}(\vec{r}))$ using the expression,

$$\Delta\rho(\vec{r}) = \frac{\rho_{PCP\,+\,guest}(\vec{r})\; - \;\left(\rho_{PCP}(\vec{r}) + \rho_{guest}(\vec{r})\right)}{n}$$

Where $\vec{r}$ is the spatial coordinate. The electron density differences obtained from periodic-DFT calculations are shown only around guest molecules in the next section; this was extracted using our in-house code that is made available on https://github.com/Nimishdwarakanath, the Cubecruncher code supplied with CP2K and the Visual Molecular Dynamics (VMD)[22] software.

Results:

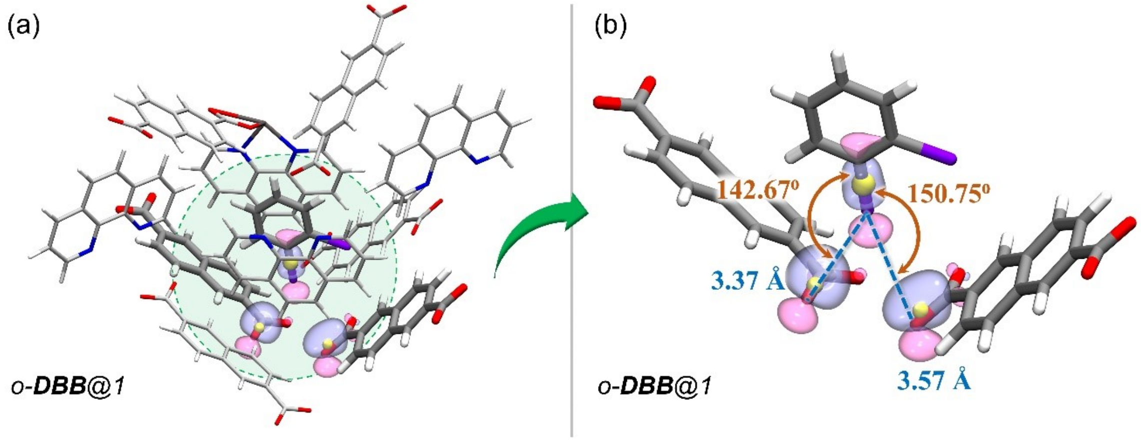

Fig S25: Reproduction of Fig. 5a with maximally localised Wannier functions (MLWFs) (violet and magenta surfaces) and maximally localised Wannier function centers (MLWFCs) (yellow spheres) for C–Br σ-bond (of o-DBB) and electron- pair in π-bonds (PB) of the framework C=O1 bonds. Violet and magenta surfaces represent MLWFs of opposite phases with isovalue of 5.0×10^{-2} a.u. with an exception of σ-bond of C–Br which is shown for an isovalue of 1.5×10^{-1} a.u. ∠C–Br· · · PB are 142.67˚ and 150.75˚ confirm that the electrons of the π-bond indeed interact with the σ-hole of bromine atom, demonstrating the prevalence of halogen bonding interactions.

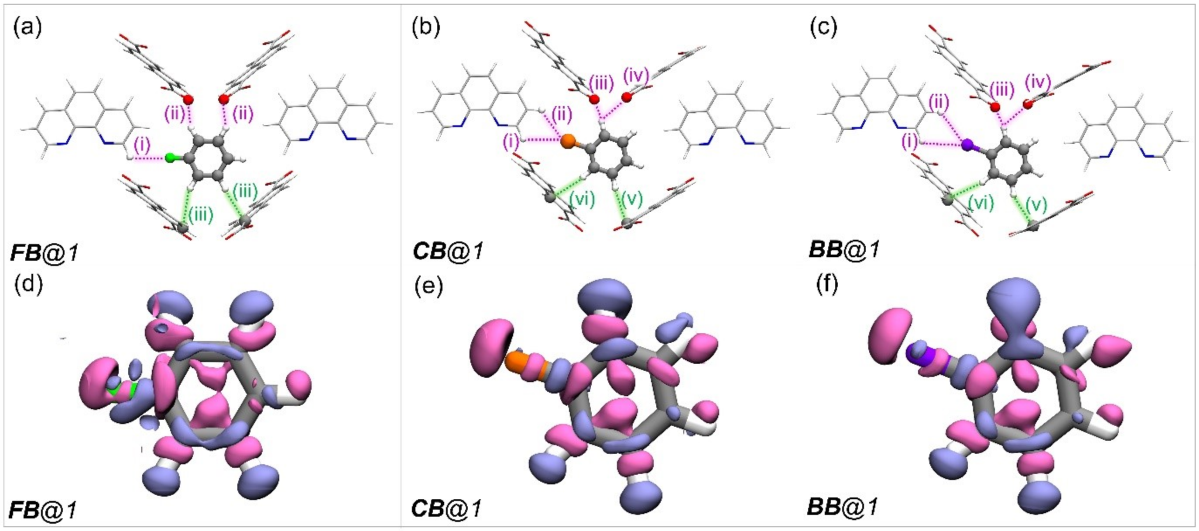

Fig S26: The guest encapsulated cavities of (a) ***FB***@1, (b) ***CB***@1, and (c) ***BB***@1, respectively. o-phen rings sandwiching the guest molecules are omitted for clarity. The colored dashed lines represent the type of supramolecular interactions--green and magenta lines represent C-H· · · π and hydrogen bonding interactions, respectively. Details of atom names, bond lengths, and angles associated with each interaction are shown in **Table S21**. (d-f) Shows the electron density differences in the vicinity of the guest in a cavity of (a) ***FB***@1, (b) ***CB***@1, and (c) ***BB***@1, respectively. Violet and magenta surfaces represent electron density gain and loss of magnitude 5.0×10^{-4} a.u., respectively. The guest encapsulated cavities shown here were obtained from the guest@1 supercells optimized using periodic DFT. The color scheme for the atoms is identical to that in Fig. 2.

Table S17: PCPs encapsulating different guests (guest@1): comparison of box parameters for the crystal structures (row called 'crystal') and the corresponding periodic-DFT-based cell optimized (row called 'CO') supercells. Error percentage in cell optimized supercell volumes (ΔV) and the corresponding binding energies (BE) per guest molecule are also presented.

		guest@1		a	b	c	α	β	γ	ΔV	BE
				(Å)			(˚)			(%)	kJ/mol
DXB	***DFB***	*o*-***DFB***	Crystal	15.12	19.71	16.85	90.00	94.06	90.00	0.48	-108.60
			CO	15.24	19.93	16.64	89.98	95.14	90.01		
		m-***DFB***	Crystal	15.12	19.71	16.85	90.00	94.06	90.00	0.57	-124.70
			CO	15.24	19.93	16.64	89.98	95.14	90.01		
		p-***DFB***	Crystal	15.77	19.95	16.02	90.00	93.43	90.00	0.67	-124.90
			CO	15.65	19.93	16.28	90.00	93.89	90.00		
	DCB	*o*-***DCB***	Crystal	15.03	21.91	15.93	90.00	90.00	90.00	0.62	-134.40
			CO	14.77	21.64	16.51	90.00	90.00	90.00		
		m-***DCB***	Crystal	15.10	19.51	17.18	90.00	91.30	90.00	-0.04	-142.70
			CO	14.87	19.48	17.47	90.00	90.35	90.00		
		p-***DCB***	Crystal	15.27	19.77	17.06	90.00	90.08	90.00	-0.22	-144.00
			CO	15.18	19.67	17.22	90.00	89.39	90.00		
	DBB	*o*-***DBB***	Crystal	15.63	22.12	15.80	90.00	90.00	90.00	1.41	-138.50
			CO	15.51	21.76	16.41	90.68	90.00	90.00		
		m-***DBB***	Crystal	15.11	19.41	17.39	90.00	92.21	90.00	-0.43	-150.70
			CO	14.82	19.40	17.66	90.00	91.43	90.00		
		p-***DBB***	Crystal	15.24	19.66	17.42	90.00	91.11	90.00	-0.30	-151.90
			CO	15.17	19.65	17.47	90.00	90.42	90.00		
XB		***FB***	Crystal	15.88	19.77	16.07	90.00	93.82	90.00	0.17	-116.03
			CO	15.73	19.97	16.10	90.00	94.65	90.09		
		CB	Crystal	7.78	19.66	16.52	90.00	91.55	90.00	-0.34	-126.06
			CO	7.66	19.55	16.83	90.13	92.13	90.01		
		BB	Crystal	7.79	19.47	16.62	90.00	91.40	90.00	-0.08	-129.78
			CO	7.70	19.27	16.99	90.21	91.74	90.05		

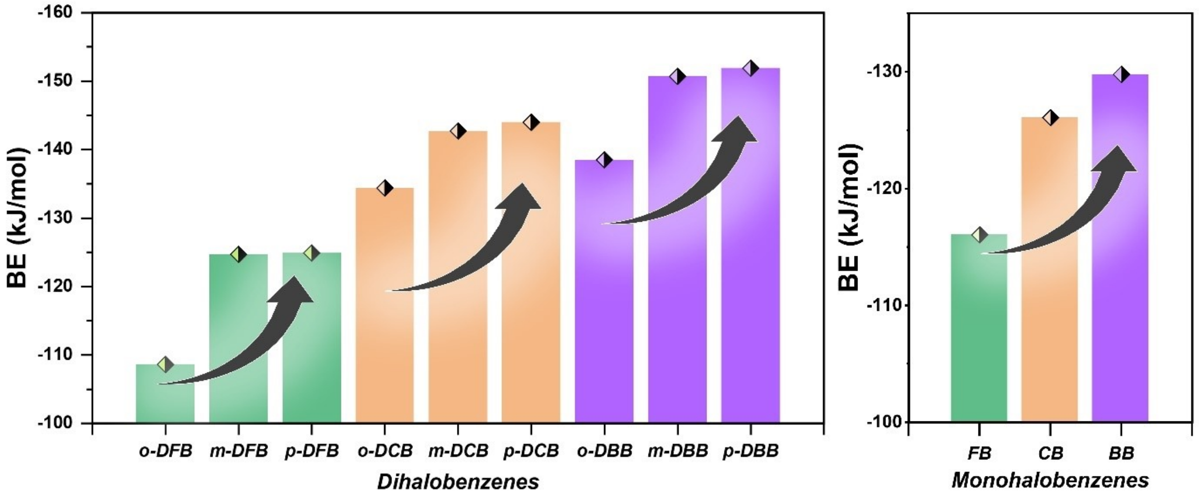

Fig. S27: DFT calculated binding energy vs. dihalobenzenes and monohalobenzenes.

Table S18: Details of specific interactions between difluorobenzene (DFB) isomers and framework indicated in Fig. 3(a-c). The nature of interactions and comparison of relevant distances and angles measured from crystal structures and cell optimized (CO) supercells are presented with the same Roman numerals used in Fig. 3(a-c). The average and standard deviation in distances/angles were calculated for eight guests of the cell optimized supercells.

ID.	Nature of Interactions	Distance(Å)		Angle (˚)	
		Crystal structure	CO structure	Crystal structure	CO structure
o-DFB					
(i)	CH···π	H6···C17		C6–H6···C17	
		3.16±0.00	3.03±0.02	165.73±0.0	162.27±0.30
(ii)	CH···π	H5···C20		C5–H5···C20	
		2.74±0.00	2.67±0.01	158.56±0.0	167.44±0.80
(iii)	CH···π	H3···C23		C3–H3···C23	
		3.19±0.00	3.19±0.02	175.60±0.00	166.49±0.77
(iv)	HB	H26···F2		C26–H26···F2	
		2.33±0.00	2.48±0.0	172.66±0.00	135.91±1.19
m-DFB					
(i)	CH···π	H14···C4		C14–H14···C4	
		2.97±0.00	2.80±0.02	156.64±0.00	155.23±0.41
(ii)	HB	H13···O2		C13–H13···O2	
		3.95±0.00	3.66±0.03	147.15±0.00	146.09±0.28
(iii)	HB	H16···O2		C16–H16···O2	
		2.92±0.00	2.69±0.03	175.60±0.00	166.49±0.77
(iv)	HB	H8···F1		C8–H8···F1	
		2.58±0.00	2.56±0.02	154.41±0.00	155.58±0.89
p-DFB					
(i)	CH···π	H3···C11		C3–H3···C11	
		2.95±0.00	2.80±0.05	176.07±0.00	174.81±0.30
(ii)	HB	H1···O1		C1–H1···O1	
		2.77±0.00	2.56±0.07	135.67±0.00	132.12±0.34
(iii)	HB	H4···F00E		C4–H4···F00E	
		2.99±0.00	2.91±0.00	124.47±0.00	117.82±1.92
(iv)	HB	H5···F00E		C5–H5···F00E	
		2.87±0.00	2.70±0.08	128.62±0.00	127.81±1.22

Table S19: Details of specific interactions between dichlorobenzene (DCB) isomers and framework indicated in Fig. 4(a-c). The nature of interactions and comparison of relevant distances and angles measured from crystal structures and cell optimized (CO) supercells are presented with the same Roman numerals used in Figure Fig. 4(a-c). The average and standard deviation in distances/angles were calculated for eight guests of the cell optimized supercells.

ID.	Nature of Interactions	Distance(Å)		Angle (˚)	
		Crystal structure	CO structure	Crystal structure	CO structure
o-DCB					
(i)	XB	Cl···O1		C–Cl···O1	
		4.11±0.00	4.09±0.02	151.76±0.00	143.83±2.27
(ii)	CH···π	H···C8		C–H···C8	
		3.04±0.00	3.35±0.29	158.37±0.00	149.84±4.84
(iii)	HB	H···O1		C–H···O1	
		3.04±0.00	3.35±0.29	131.07±0.00	126.26±9.93
m-DCB					
(i)	CH···π	H15···C5		C15–H15···C5	
		2.89±0.00	2.74±0.01	157.77±0.00	156.48±0.01
(ii)	HB	H16···O1		C16–H16···O1	
		3.68±0.00	3.49±0.00	147.36±0.00	146.82±0.01
(iii)	HB	H13···O1		C13–H13···O1	
		2.84±0.00	2.65±0.00	135.60±0.00	133.81±0.02
(iv)	HB	H8···Cl1		C8–H8···Cl1	
		2.95±0.00	2.79±0.00	146.45±0.00	145.93±0.00
p-DCB					
(i)	CH···π	H15···C10		C15–H15···C10	
		3.01±0.00	2.85±0.00	160.26±0.00	159.13±0.11
(ii)	HB	H13···O1		C13–H13···O1	
		2.66±0.00	2.51±0.02	132.21±0.00	130.47±0.09
(iii)	HB	H4···Cl1		C4–H4···Cl1	
		3.13±0.00	3.02±0.00	129.89±0.00	127.69±0.00
(iv)	HB	H5···Cl1		C5–H5···Cl1	
		3.24±0.00	3.18±0.00	125.86±0.00	119.78±0.0

Table S20: Details of specific interactions between dibromobenzene (DBB) isomers and framework indicated in Fig. 5(a-c). The nature of interactions and comparison of relevant distances and angles measured from crystal structures and cell optimized (CO) supercells are presented with the same Roman numerals used in Fig. 5(a-c). The average and standard deviation in distances/angles were calculated for eight guests of the cell optimized supercells.

ID.	Nature of Interactions	Distance(Å)		Angle (˚)	
		Crystal structure	CO structure	Crystal structure	CO structure
o-DBB					
(i)	XB	Br···O1		C–Br···O1	
		3.98±0.0	3.86±0.04	157.33±0.0	157.42±0.12
(ii)	XB	Br···C4		C–Br···C4	
		3.80±0.00	3.94±0.00	176.39±0.00	163.06±0.00
(iii)	CH···π	H···C8		C–H···C8	
		3.17±0.00	3.11±0.18	153.64±0.00	162.83±4.26
(iv)	HB	H···O1		C–H···O1	
		3.11±0.00	2.91±0.00	120.52±0.00	124.17±2.36
m-DBB					
(i)	CH···π	H15···C2		C15–H15···C2	
		2.89±0.00	2.74±0.01	157.77±0.00	156.48±0.01
(ii)	HB	H16···O2		C16–H16···O2	
		3.58±0.00	3.39±0.00	146.00±0.00	145.61±0.00
(iii)	HB	H13···O2		C13–H13···O2	
		2.89±0.00	2.70±0.00	136.26±0.00	134.90±0.00
(iv)	HB	H8···Br1		C8–H8···Br1	
		3.02±0.00	2.90±0.00	143.73±0.00	141.84±0.00
p-DBB					
(i)	CH···π	H15···C10		C15–H15···C10	
		3.01±0.00	2.85±0.00	160.26±0.00	159.13±0.11
(ii)	HB	H13···O2		C13–H13···O2	
		2.66±0.00	2.51±0.02	132.21±0.00	130.47±0.09
(iii)	HB	H4···Br1		C4–H4···Br1	
		3.33±0.00	3.24±0.00	124.44±0.00	118.15±0.06
(iv)	HB	H5···Br1		C5–H5···Br1	
		3.17±0.00	3.04±0.00	130.64±0.00	127.50±0.05

Table S21: Details of specific interactions between fluorobenzene (FB), chlorobenzene (CB), and bromobenzene (BB) guests and the framework indicated in **Figure S26**. The nature of interactions and comparison of relevant distances and angles measured from crystal structures and cell optimized (CO) supercells are presented with the same Roman numerals used in **Figure S26**. The average and standard deviation in distances/angles were calculated for eight guests of the cell optimized supercells.

ID.	Nature of Interactions	Distance(Å)		Angle (˚)	
		Crystal structure	CO structure	Crystal structure	CO structure
FB					
(i)	HB	H8⋯F4		C8–H8⋯F4	
		2.69±0.00	2.67±0.03	138.33±0.0	127.32±0.04
(ii)	HB	H15⋯O2		C15–H15⋯O2	
		2.77±0.00	3.67±0.03	150.84±0.00	135.50±2.70
(iii)	CH⋯π	H13⋯C4		C13–H13⋯C4	
		2.91±0.00	2.74±0.00	179.37±0.00	176.00±1.18
CB					
(i)	HB	H18⋯Cl1		C18–H18⋯Cl1	
		3.43±0.00	3.41±0.01	122.73±0.00	117.53±0.56
(ii)	HB	H17⋯Cl1		C17–H17⋯Cl1	
		3.20±0.00	3.10±0.02	132.17±0.00	131.08±0.65
(iii)	HB	H2⋯O2		C2–H2⋯O2	
		2.77±0.00	2.53±0.02	134.02±0.00	134.02±0.49
(iv)	HB	H2⋯O1		C2–H2⋯O1	
		3.08±0.00	2.99±0.01	123.13±0.00	116.27±0.23
(v)	CH⋯π	H5⋯C29		C5–H5⋯C29	
		2.99±0.00	2.83±0.01	170.85±0.00	164.97±0.21
(vi)	CH⋯π	H6⋯C26		C6–H6⋯C26	
		3.00±0.00	2.82±0.02	171.32±0.00	168.88±0.06
BB					
(i)	HB	H12⋯Br1		C12–H12⋯Br1	
		3.53±0.00	3.59±0.06	124.48±0.00	118.62±0.35
(ii)	HB	H11⋯Br1		C11–H11⋯Br1	
		3.41±0.00	3.36±0.00	128.92±0.00	128.37±0.40
(iii)	HB	H27⋯O3		C27–H27⋯O3	
		2.82±0.00	2.56±0.05	129.95±0.00	128.92±0.88
(iv)	HB	H27⋯O2		C27–H27⋯O2	
		3.04±0.00	3.36±0.00	128.92±0.00	128.37±0.40
(v)	CH⋯π	H30⋯C18		C30–H30⋯C18	
		2.96±0.00	2.79±0.02	171.24±0.00	167.53±0.50
(vi)	CH⋯π	H31⋯C15		C31–H31⋯C15	
		3.07±0.00	2.89±0.02	169.61±0.00	167.79±0.11

References

1 R. Haldar, R. Matsuda, S. Kitagawa, S. J. George and T. K. Maji, Amine-Responsive Adaptable Nanospaces: Fluorescent Porous Coordination Polymer for Molecular Recognition, *Angew. Chemie Int. Ed.*, 2014, **53**, 11772–11777.

2 SMART (V 5.628), SAINT (V 6.45a), XPREP, SHELXTL; Bruker AXS Inc. Madison, Winsconsin, USA. 2004.

3 G. M. Sheldrick, Siemens Area Detector Absorption Correction Program, University of Götingen, Götingen, Germany. 1994.

4 A. Altomare, G. Cascarano, C. Giacovazzo, A. GuagliardiCompletion and refinement of crystal structures with SIR92, *J. Appl. Cryst.*, 1993, **26**, 343–350.

5 G. M. Sheldrick, SHELXL-97, Program for Crystal Structure Solution and Refinement; University of Götingen, Götingen, Germany. 1997.

6 A. Spek, Single-crystal structure validation with the program PLATON. Journal of Applied Crystallography. 2003, 36, 7-13.

7 G. M. Sheldrick, SHELXS 97, Program for the Solution of Crystal Structure, University Götingen, Germany. 1997.

8 L. Barbour, X-Seed 4: updates to a program for small-molecule supramolecular crystallography. Journal of Applied Crystallography. 2020, 53, 1141-1146.

9 B. G. LIPPERT and J. H. and M. PARRINELLO, A hybrid Gaussian and plane wave density functional scheme, *Mol. Phys.*, 1997, **92**, 477–488.

10 J. VandeVondele, M. Krack, F. Mohamed, M. Parrinello, T. Chassaing and J. Hutter, Quickstep: Fast and accurate density functional calculations using a mixed Gaussian and plane waves approach, *Comput. Phys. Commun.*, 2005, **167**, 103–128.

11 T. D. Kühne, M. Iannuzzi, M. Del Ben, V. V Rybkin, P. Seewald, F. Stein, T. Laino, R. Z. Khaliullin, O. Schütt, F. Schiffmann, D. Golze, J. Wilhelm, S. Chulkov, M. H. Bani-Hashemian, V. Weber, U. Borštnik, M. Taillefumier, A. S. Jakobovits, A. Lazzaro, H. Pabst, T. Müller, R. Schade, M. Guidon, S. Andermatt, N. Holmberg, G. K. Schenter, A. Hehn, A. Bussy, F. Belleflamme, G. Tabacchi, A. Glöß, M. Lass, I. Bethune, C. J. Mundy, C. Plessl, M. Watkins, J. VandeVondele, M. Krack and J. Hutter, CP2K: An electronic structure and molecular dynamics software package - Quickstep: Efficient and accurate electronic structure calculations, *J. Chem. Phys.*, 2020, **152**, 194103.

12 J. Hutter, M. Iannuzzi, F. Schiffmann and J. VandeVondele, cp2k: atomistic simulations of condensed matter systems, *WIREs Comput. Mol. Sci.*, 2014, **4**, 15–25.

13 J. P. Perdew, K. Burke and M. Ernzerhof, Generalized Gradient Approximation Made Simple, *Phys. Rev. Lett.*, 1996, **77**, 3865–3868.

14 S. Grimme, J. Antony, S. Ehrlich and H. Krieg, A consistent and accurate ab initio parametrization of density functional dispersion correction (DFT-D) for the 94 elements H-Pu, *J. Chem. Phys.*, 2010, **132**, 154104.

15 S. Grimme, S. Ehrlich and L. Goerigk, Effect of the damping function in dispersion corrected

density functional theory, *J. Comput. Chem.*, 2011, **32**, 1456–1465.

16 M. Krack, Pseudopotentials for H to Kr optimized for gradient-corrected exchange-correlation functionals, *Theor. Chem. Acc.*, 2005, **114**, 145–152.

17 S. Goedecker, M. Teter and J. Hutter, Separable dual-space Gaussian pseudopotentials, *Phys. Rev. B*, 1996, **54**, 1703–1710.

18 C. Hartwigsen, S. Goedecker and J. Hutter, Relativistic separable dual-space Gaussian pseudopotentials from H to Rn, *Phys. Rev. B*, 1998, **58**, 3641–3662.

19 P. E. Blöchl, Electrostatic decoupling of periodic images of plane-wave-expanded densities and derived atomic point charges, *J. Chem. Phys.*, 1995, **103**, 7422–7428.

20 G. J. Martyna and M. E. Tuckerman, A reciprocal space based method for treating long range interactions in ab initio and force-field-based calculations in clusters, *J. Chem. Phys.*, 1999, **110**, 2810–2821.

21 L. Genovese, T. Deutsch, A. Neelov, S. Goedecker and G. Beylkin, Efficient solution of Poisson's equation with free boundary conditions, *J. Chem. Phys.*, 2006, **125**, 74105.

22 L. Genovese, T. Deutsch and S. Goedecker, Efficient and accurate three-dimensional Poisson solver for surface problems, *J. Chem. Phys.*, 2007, **127**, 54704.
